# Breeding *Alnus* species for resistance to *Phytophthora* disease in the Iberian Peninsula

**DOI:** 10.3389/fpls.2024.1499185

**Published:** 2024-12-09

**Authors:** Daniela Cordeiro, Alberto Pizarro, M. Dolores Vélez, M. Ángeles Guevara, Nuria de María, Paula Ramos, Irene Cobo-Simón, Alba Diez-Galán, Alfredo Benavente, Verónica Ferreira, M. Ángela Martín, Patricia M. Rodríguez-González, Alejandro Solla, M. Teresa Cervera, Julio Javier Diez-Casero, José Antonio Cabezas, Carmen Díaz-Sala

**Affiliations:** ^1^ Departamento de Ciencias de la Vida, Facultad de Ciencias, Universidad de Alcalá, Madrid, Spain; ^2^ Departamento de Ecología y Genética Forestal, Instituto de Ciencias Forestales (ICIFOR), Instituto Nacional de Investigación y Tecnología Agraria y Alimentaria - Consejo Superior de Investigaciones Científicas (ICIFOR-INIA, CSIC), Madrid, Spain; ^3^ Instituto Universitario de Investigación en Gestión Forestal Sostenible (iuFOR), Universidad de Valladolid, Palencia, Spain; ^4^ Departamento de Producción Vegetal y Recursos Forestales, Escuela Técnica Superior de Ingenierías Agrarias (ETSIIAA), Universidad de Valladolid, Palencia, Spain; ^5^ MARE – Marine and Environmental Sciences Centre, ARNET – Aquatic Research Network, Department of Life Sciences, University of Coimbra, Coimbra, Portugal; ^6^ Departamento de Genética, Escuela Técnica Superior de Ingeniería Agronómica y de Montes (ETSIAM), Universidad de Córdoba, Córdoba, Spain; ^7^ Forest Research Centre, Associate Laboratory TERRA, School of Agriculture, University of Lisbon, Lisbon, Portugal; ^8^ Ingeniería Forestal y Medio Natural, Centro Universitario de Plasencia, Instituto Universitario de Investigación de la Dehesa (INDEHESA), Universidad de Extremadura, Plasencia, Spain

**Keywords:** alder decline, environmentally friendly management, forest diseases, forest trees, oomycetes, riparian ecosystems

## Abstract

Alders are widely distributed riparian trees in Europe, North Africa and Western Asia. Recently, a strong reduction of alder stands has been detected in Europe due to infection by *Phytophthora* species (Stramenopila kingdom). This infection causes a disease known as alder dieback, characterized by leaf yellowing, dieback of branches, increased fruit production, and bark necrosis in the collar and basal part of the stem. In the Iberian Peninsula, the drastic alder decline has been confirmed in the Spanish Ulla and Ebro basins, the Portuguese Mondego and Sado basins and the Northern and Western transboundary hydrographic basins of Miño and Sil, Limia, Douro and Tagus. The damaging effects of alder decline require management solutions that promote forest resilience while keeping genetic diversity. Breeding programs involve phenotypic selection of asymptomatic individuals in populations where severe damage is observed, confirmation of tree resistance via inoculation trials under controlled conditions, vegetative propagation of selected trees, further planting and assessment in areas with high disease pressure and different environmental conditions and conservation of germplasm of tolerant genotypes for reforestation. In this way, forest biotechnology provides essential tools for the conservation and sustainable management of forest genetic resources, including material characterization for tolerance, propagation for conservation purposes, and genetic resource traceability, as well as identification and characterization of *Phytophthora* species. The advancement of biotechnological techniques enables improved monitoring and management of natural resources by studying genetic variability and function through molecular biology methods. In addition, *in vitro* culture techniques make possible large-scale plant propagation and long-term conservation within breeding programs to preserve selected outstanding genotypes.

## Introduction

Alders are deciduous riparian trees distributed mostly in the Mediterranean, temperate and boreal zones of the Northern Hemisphere (Bjelke et al., 2016) up to the Himalayas and the Andes. These trees compose the genus *Alnus* (Family Betulaceae), represented by more than 40 species. In the Iberian Peninsula, two different species coexist: *Alnus glutinosa* (L.) Gaertn. (common name: common alder) and *Alnus lusitanica* Vít, Douda & Mandák (common name: Iberian alder; Vít et al., 2017). Common alder is the most widespread alder species in Europe and Western Asia, playing a significant ecological role as a key component of the riparian vegetation along streams (Claessens et al., 2010; Smeriglio et al., 2022). However, in the Iberian Peninsula, the presence of *A. glutinosa* is limited to the northeast region. Conversely, *A. lusitanica* is the most representative and widespread alder species with a large distribution in the northwest region (Gomes Marques et al., 2024a; Martín et al., 2024).

Alders are actinorhizal plants that fix atmospheric nitrogen, therefore contributing very significantly to nitrogen dynamics at the local and landscape scales. In addition, they stabilize streams and riverbanks, functioning as a protective barrier against flooding, preventing waterlogging of crops and surrounding areas, and mitigating the damage caused after periods of widespread rainfall. Also, alders contribute to the rapid colonization of abandoned sites and the maintenance of biodiversity by providing refuge for terrestrial and aquatic organisms (Compton et al., 2003; Wipfli and Musslewhite, 2004; Claessens et al., 2010; Handa et al., 2014). This combination of attributes, along with the provision and cultural ecosystem services that are derived from streams and rivers, makes alder replacement by other species very difficult.

Over the recent decades, different alder species have been severely impacted by decline and mortality events caused by abiotic factors, such as extended periods of drought followed by flooding, as well as biotic factors (Ferreira et al., 2022; Gomes Marques et al., 2022), which accumulate to already existing long-term pressures on rivers (Vörösmarty et al., 2010). Along with global change, an increase in outbreaks of invasive pathogenic fungi and oomycetes has severely affected native plants worldwide (Fisher et al., 2020; Gomes Marques et al., 2024b).

Recently, a strong reduction of alder stands has been detected in Europe due to infection by the *Phytophthora alni* species complex Brasier & S.A. Kirk (Bjelke et al., 2016). This complex includes a group of pathogens that cause *Phytophthora* disease of alder, also known as alder dieback, affecting different organs of the aerial part of the tree and roots (Gibbs et al., 1999). Although *P. ×alni* (Husson et al., 2015) is the most aggressive species within the complex, a possible synergy between *Phytophthora* species in the damage caused to alders is unknown. The aggressiveness is favored by mild winters and warm, but not too hot, summers (Bjelke et al., 2016; Gomes Marques et al., 2024b; Horta Jung et al., 2024). Also, global environmental changes may promote shifts in the pathogen distribution and impact. Their dispersal occurs primarily along water currents which transport thousands of infective zoospores, thus constituting an important route of dissemination of *Phytophthora* species. Due to the dendritic structure of river networks, *Phytophthora* can spread rapidly to new areas, notably downstream (Bjelke et al., 2016). The *Phytophthora* zoospores usually infect the host through the root system, mainly fine roots, or by wounds at the base of the trunk and ascend through its tissues causing lesions in the cambium (Brasier and Kirk, 2001; Černý and Strnadová, 2012). Thus, alders may be subjected to multiple infections over time due to their proximity to the river and the contact of their tissues with surface runoff. Therefore, changes in phytosanitary state and vigor, as well as the degree of tree survival over time are conditioned by environmental factors, including the concentration of inoculum in the soil, the soil type, the water flow velocity, and the geomorphic position of the tree in relation to water (Gomes Marques et al., 2024b).

The wide distribution of alders, with isolated local populations, has resulted in a high genetic diversity that allows them to respond differently to selection pressures related to stand structure (like canopy composition and density) and edaphic, abiotic and biotic factors. In the same way, it has also resulted in inbreeding and moderate local differentiation, partly associated with the ease of seed dispersal through river channels (Štochlová et al., 2012). Given the local adaptation of alders, the identification/selection of more tolerant or resistant genotypes in distant stretches of the same river should seek to retain the adaptive traits so that they can thrive in different environmental conditions. Even when infected, some alders may look asymptomatic and can remain so for a long time (Elegbede et al., 2010). This would also contribute to broadening the genetic base of resistance, thereby reducing the risk of the pathogen overcoming resistance (Sniezko and Koch, 2017). For these reasons, it is very important to understand and protect the existing diversity and to identify resistant genotypes with as much genetic variation as possible and from suitable sources, so they can be used for riverbank restoration.

In this review, the impacts of alder dieback in the Iberian Peninsula and the potential breeding strategies for alder resistance to *Phytophthora* are summarized. New approaches to improve the resistance selection process and breeding are also described.

## Alder genetic diversity

During the last two decades, several studies have used molecular markers to address the genetic diversity and structure of European alders (Mingeot et al., 2010; De Kort et al., 2014a, De Kort et al., 2014b; Beatty et al., 2015; Cubry et al., 2015; Havrdová et al., 2015; Gryta et al., 2017). Although traditionally considered diploid species, recent studies using cytometry and molecular markers revealed variation in ploidy level (Lepais et al., 2013; Mandák et al., 2016). This way, Mandák et al. (2016) identified three main groups across Europe in what was before considered to be *A. glutinosa*. In addition to the most common and widely distributed diploid (2n = 2x = 28) *A. glutinosa*, two tetraploid (2n = 4x = 56) clusters were identified: one in western Balkan Peninsula (*A. rohlenae* Vít, Douda & Mandák) and the other in the Iberian Peninsula and North Africa (*A. lusitanica*) (Vít et al., 2017; Gomes Marques et al., 2024a). The proposed post-glacial recolonization of Europe by alders would have taken place from multiple refuges in the north of the Iberian, Apennine and Balkan Peninsulas, composed of diploid populations (*A. glutinosa*), with no involvement of tetraploid populations, which may have originated later (Comes and Kadereit, 1998; Havrdová et al., 2015; Mandák et al., 2016; Šmíd et al., 2020).

**Figure 1:**
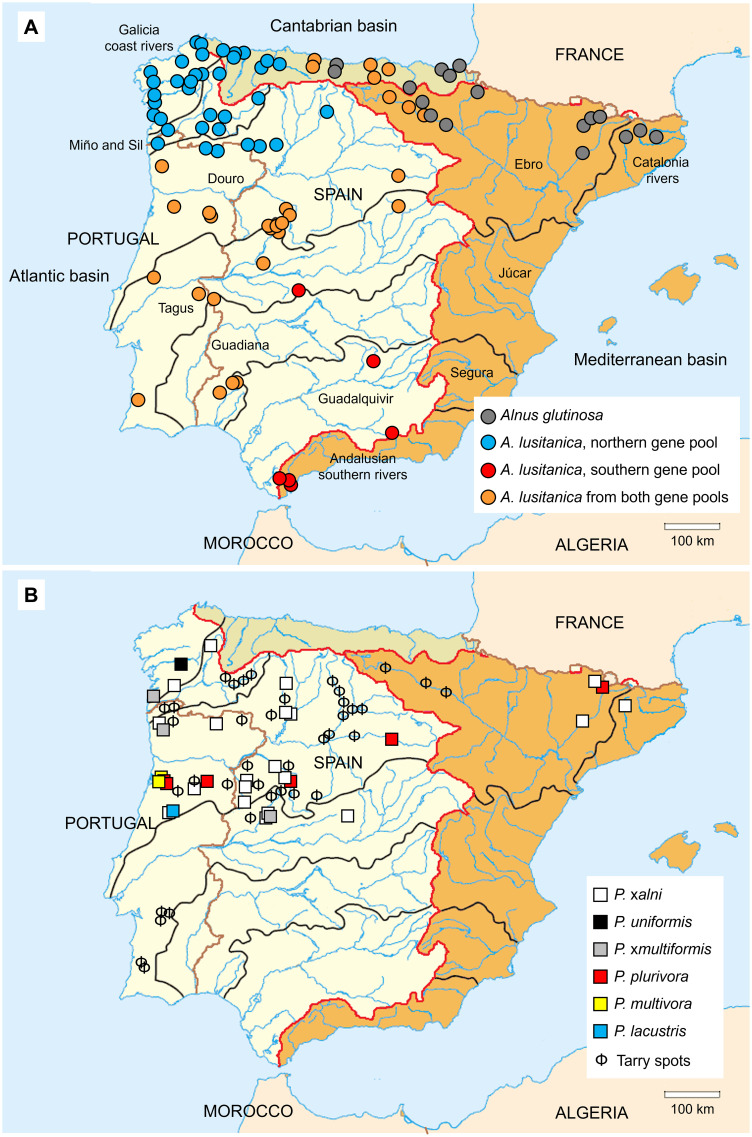
**(A)** Distribution of *Alnus* species within the Iberian Peninsula according to ploidy assessment (Havrdová et al., 2015; Vít et al., 2017; Gomes Marques et al., 2022; Sanna et al., 2023) and to genetic structure analyses by using microsatellite markers (Gomes Marques et al., 2024a; Martín et al., 2024). **(B)** Location of *Phytophthora* species isolated from unhealthy alder trees (Solla et al., 2010; Pintos-Varela et al., 2010, 2012, 2016; Haque et al., 2014; Jung et al., 2016; Kanoun-Boulé et al., 2016; Ferreira et al., 2022; Bregant et al., 2023; Vieites-Blanco et al., 2023; Gomes Marques et al., 2024b; Gomes Marques, 2024) and stands with characteristic tarry spots on alder trunks from which *Phytophthora* was not isolated yet. Edges of major river basins are highlighted in red and black, main rivers in blue and country borders in brown.

In the Iberian Peninsula, the Iberian alder (*A. lusitanica*) and the common alder (*A. glutinosa*) are native tree species occurring in riparian and wetland forest communities. Both species contribute to shaping the characteristics, communities and functioning of stream ecosystems (**Table 1**). The most representative and widespread alder is the tetraploid *A. lusitanica*, which is widespread from Morocco, and shows three main genetic groups with a clear geographical distribution in the northern, western and central and southern regions (**Figure 1A**; Martín et al., 2024), under Atlantic and continental climates. The common alder is present in the Ebro basin and some northeast Cantabrian and Catalonian basins (**Figure 1A**). The Ebro River represents the northeast limit for the distribution of this species (Sanna et al., 2023; Martín et al., 2024), but also a contact zone between *A. lusitanica* and *A. glutinosa*. Even so, to date, there is no evidence of gene flow between these species, as evidenced by the absence of triploid individuals among the samples prospected in this area (Martín et al., 2024). In turn, although rare, in the Balkans, triploid individuals have been identified in the overlapping distribution areas of *A. glutinosa* and the tetraploid *A. rohlenae* (Mandák et al., 2016; Šmíd et al., 2020). Based on these findings, future studies might also identify triploids in the area where *A. lusitanica* and *A. glutinosa* meet in the Iberian Peninsula.

**Table 1:** Summary of alder tree traits and their contributions to stream ecosystems.

Alder trait	Contribution to streams	References
**Extensive root system**	Bank stabilization and channel morphology	Claessens et al., 2010
**Woody adventitious roots**	Habitat, refuge and feeding ground for aquatic organisms	Claessens et al., 2010
**Nitrogen-fixing owing to the symbiotic association with *Frankia alni* in root nodules**	Increase of Nitrogen concentrations in water through Nitrogen export from Nitrogen-enriched soil	Compton et al., 2003; Shaftel et al., 2012
**Large canopy**	Shade in summer	Bjelke et al., 2016
	Habitat and refuge for aquatic organisms during their terrestrial life stage	Bjelke et al., 2016
	Habitat, refuge, food and feeding ground for terrestrial organisms, which in turn may fall into streams being food for aquatic organisms	Bjelke et al., 2016
**Autumn leaf litterfall**	Supply of leaf litter in autumn, allowing the early instars of aquatic insect detritivores to have a high-quality food resource	Pozo et al., 1997
**Leaf litter with high concentrations of Nitrogen and low concentrations of recalcitrant carbon**	High-quality and fast-decomposing leaf litter	Feio et al., 2010; Woodward et al., 2012
	Faster decomposition of more recalcitrant leaf litter from other tree species	Ferreira et al., 2012; Alonso et al., 2021; Rubio-Ríos et al., 2023

## Ecological importance of *Alnus lusitanica* and *Alnus glutinosa* in Iberian riparian and wetland ecosystems

According to the European Habitats Directive 92/43/EEC, alders are considered key components of alluvial forests, which are priority habitats for biodiversity conservation (priority habitat 91E0*). Indeed, alders are trees that live close to water and so have high ecological importance (**Table 1**).

**Figure 2:**
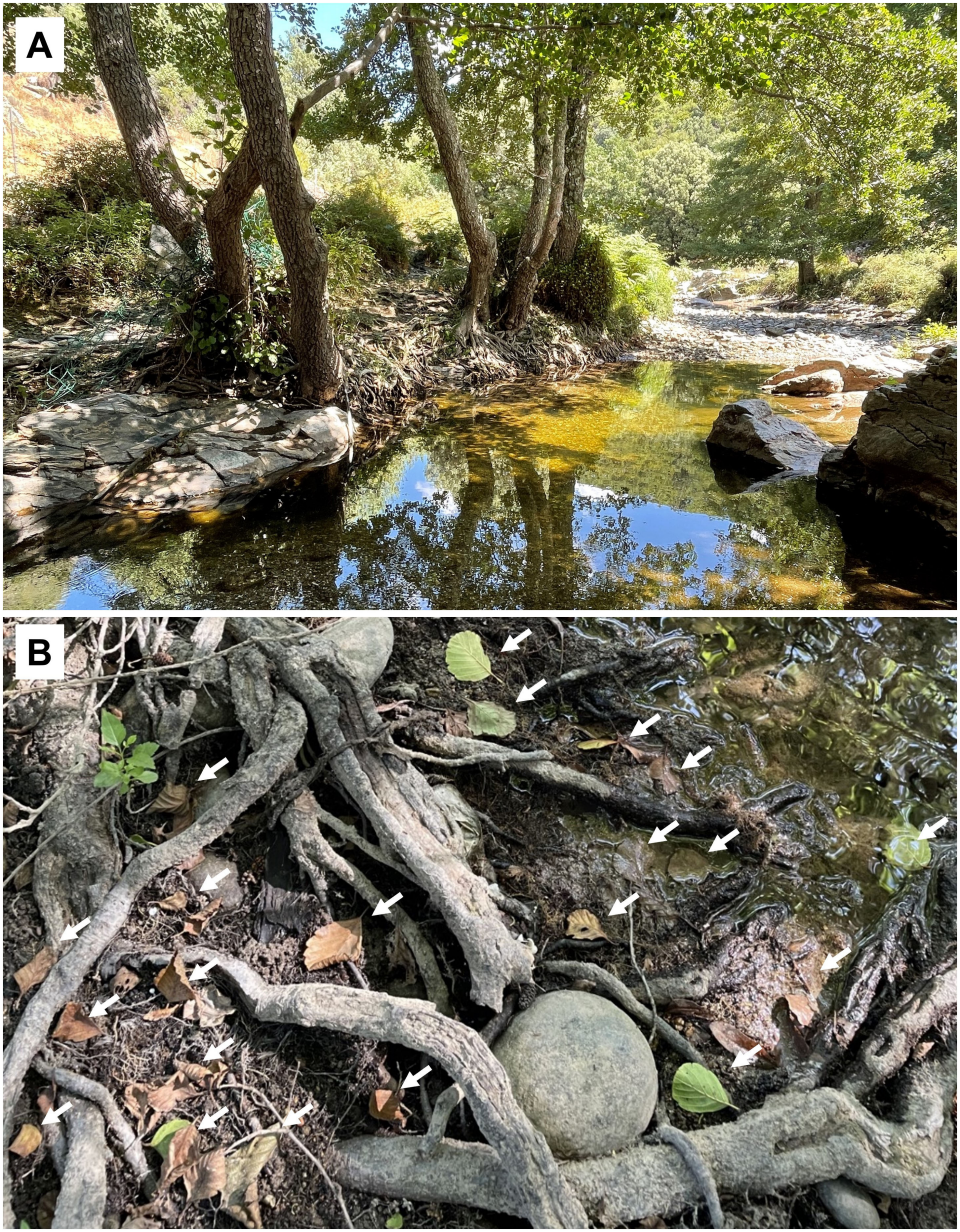
**(A)** *Alnus glutinosa* trees provide streambank stabilization. **(B)** Leaf litter of *A*. *lusitanica*, indicated by arrows. Both species show root systems that tolerate submersion, with long tap roots that ensure anchorage during water level decline.

The extensive root system of alders contributes to defining the morphology of the stream channel, thus creating important habitats for other organisms (**Figure 2A**). Moreover, woody adventitious roots provide habitat, refuge and feeding grounds for aquatic organisms (Claessens et al., 2010). Their root system also provides an important ecosystem service by contributing to stabilize riverbanks. Alders are also nitrogen-fixing trees owing to their symbiotic association with the nitrogen-fixing bacteria *Frankia alni* (Voronin) Von Tubeuf in root nodules, which increases soil nitrogen availability (Teklehaimanot and Mmolotsi, 2007; Claessens et al., 2010). As a result, alder trees in the watershed could significantly increase nitrogen levels in streams. This was verified in the USA, where redder alder (*A. rubra* Bong.) cover in the watershed was linked to higher nitrogen concentrations in stream water (Compton et al., 2003; Shaftel et al., 2012). Increases in soil nitrogen availability in the presence of alder may also increase the nutrient concentrations of leaf litter of non-nitrogen-fixing species (Shainsky and Rose, 1995; Rhoades et al., 2001).

Alder trees do not tolerate shade, making them pioneer species, and the choice to help restore degraded riparian habitats. The shade provided by these trees during summer limits instream primary production while contributes to keeping stream water cool (Bjelke et al., 2016). Furthermore, the alder canopy provides habitat, refuge, food and feeding grounds to terrestrial and aquatic species during their terrestrial life stage, which may provide food inputs to the stream ecosystem when falling into the water (Wipfli, 1997).

Moreover, leaf litter of Iberian and common alders (**Figure 2B**) is soft and has high nitrogen concentration and low concentration of recalcitrant carbon, in comparison with leaf litter of other Iberian native tree species (Jabiol et al., 2019; Ferreira et al., 2022). This makes it a very palatable food resource for aquatic microbial decomposers and macroinvertebrate detritivores, which prefer alder leaf litter to more recalcitrant one (Graça and Cressa, 2010; Alonso et al., 2021), resulting in its fast decomposition (Feio et al., 2010; Woodward et al., 2012). Especially in autumn, alder leaf litterfall offers an important food supply to aquatic food webs (Pozo et al., 1997), providing a high-quality food resource for the early stages of detritivores aquatic insects (Molinero and Pozo, 2006). In addition, alder leaf litter often stimulates the decomposition of litter mixtures (Alonso et al., 2021), not only because it is a fast-decomposing leaf litter itself (Rubio-Ríos et al., 2023), but also because it stimulates the decomposition of more recalcitrant leaf litter due to the likely reduction in nutrient limitation (Ferreira et al., 2012; Alonso et al., 2024).

## Alder *Phytophthora* species in Spain and Portugal

Riparian forests are particularly vulnerable to pathogen invasions, which spread rapidly along rivers, taking advantage of the current and becoming more effective once they reach stagnant waters (Bjelke et al., 2016). Since the 1990s, the principal pathogens associated with alder disease have been the oomycete of the genus *Phytophthora*. The first observation of the pathogen infecting alder trees in Europe was in the UK in 1993 (Brasier et al., 1995), and it rapidly spread being quickly detected in a large part of Europe (Brasier et al., 2022). Although the mechanisms behind the rapid spread of the pathogen across Europe are not well understood, it is known that water enhances the pathogen’s sporulation, spread and infection via zoospores (Chen et al., 2022). Furthermore, other authors consider that human activities have significantly contributed to the rapid spread of the disease. For instance, plants already infected by *Phytophthora* from nurseries may have been used for the reforestation of riparian forests, allowing the introduction of new pathogens (Gibbs et al., 2003; Eschen et al., 2015; Jung et al., 2016; Tremblay et al., 2018; Mora-Sala et al., 2022).

**Figure 3:**
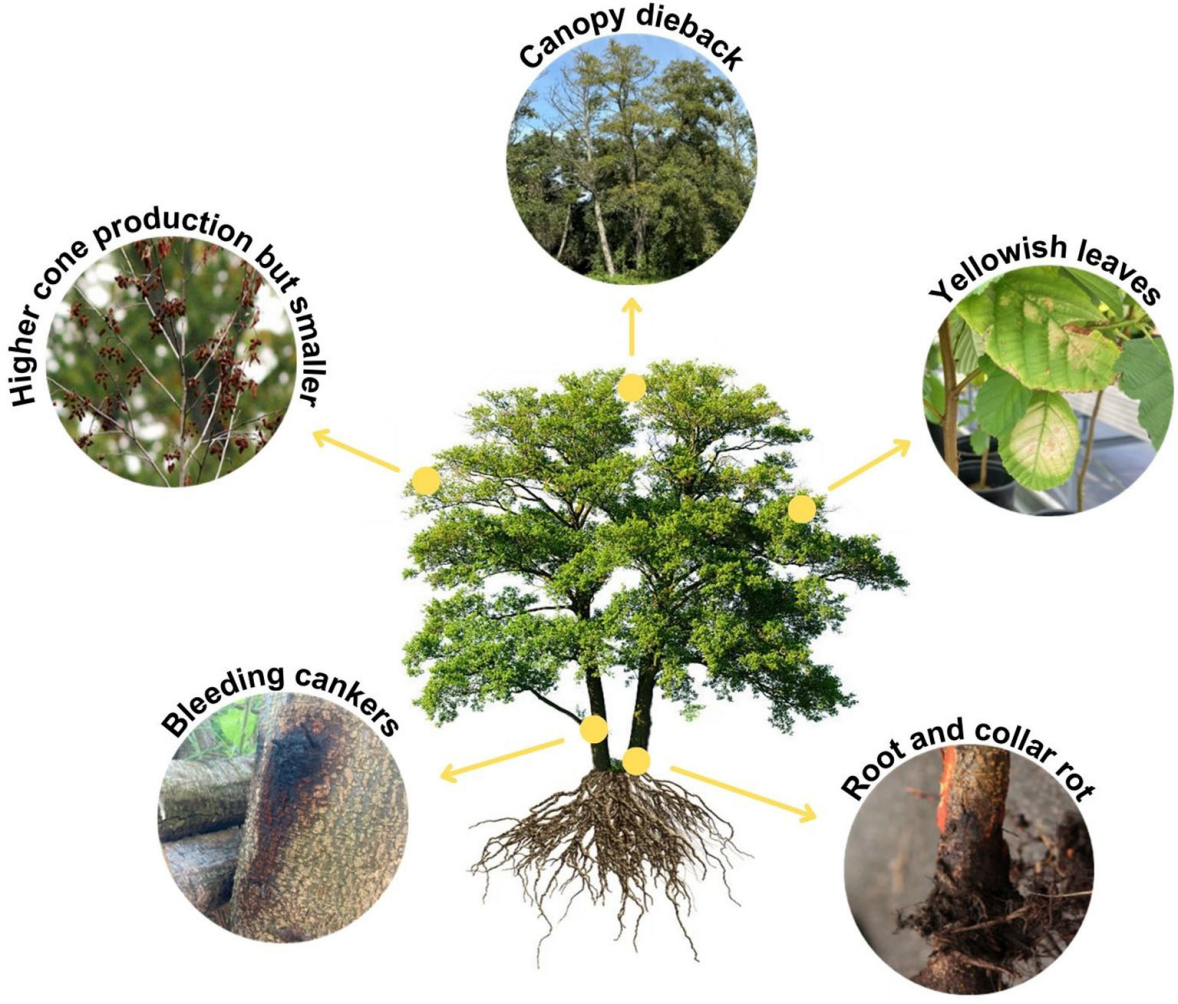
Representative scheme of the main symptoms observed in alder trees infected by different *Phytophthora* species.

In the Iberian Peninsula, the first reports of outbreaks of *Phytophthora* associated with alders date from 2009 in Spain and from 2016 in Portugal (Pintos-Varela et al., 2010; Solla et al., 2010; Kanoun-Boulé et al., 2016). The main pathogens causing the disease in alder trees are grouped in the *P. alni* complex, which includes *P. ×alni*, *P. uniformis* and *P. ×multiformis*, described as species by Husson et al. (2015) (Bjelke et al., 2016; Nave et al., 2021; Trzewik et al., 2021; Bregant et al., 2023). Over the years, new species of *Phytophthora* (**Table 2**; **Figure 1B**) have been detected and isolated from diseased alders presenting symptoms similar to the disease caused by *P. alni* complex infection (**Figure 3**). In particular, *P. plurivora* Jung & Burgess (Jung and Burgess, 2009) has been isolated from bark cankers on several occasions, being the second most isolated species in the Iberian Peninsula (Vieites-Blanco et al., 2023).

**Table 2:** First reported distribution of different species of *Phytophthora* associated with alder in Spain.

Species	River and Spanish region	References
***Phytophthora ×alni***	Avia River – Galicia Miño River – Galicia	Pintos-Varela et al., 2010; Solla et al., 2010
***P. uniformis***	Deza River – Galicia	Pintos-Varela et al., 2012
***P. plurivora***	Tera River – Castile and León Tormes River – Castile and León	Haque et al., 2014
***P. hydropathica***	Arnoia and Avia Rivers – Galicia	Pintos-Varela et al., 2016
***P. ×multiformis***	Muiños River – Galicia	Pintos-Varela et al., 2017
***P. lacustris***	Miño-Sil – Galicia and León	Rial-Martínez et al., 2023

All these *Phytophthora* taxa (**Table 2**) generate common symptoms in alders (**Figure 3**) including small and yellowish leaves, increased cone production but cones smaller than the ones from healthy alders, canopy dieback, growth reduction, bark necrosis, bleeding cankers, exudations in the collar and basal part of the stem, root and collar rot, and tree mortality (Bjelke et al., 2016; Corcobado et al., 2023; Handa et al., 2014; Redondo et al., 2015a). Nonetheless, the pathogenicity of the different *Phytophthora* species varies. Different methods have been described to study the pathogenicity of *Phytophthora* species under controlled conditions (Haque et al., 2015; Chandelier et al., 2016). Within the *P. alni* complex*, P. ×alni* is considered the most aggressive (Haque et al., 2015). However, studies comparing the pathogenicity between *P. plurivora* and *P. ×alni* (the most pathogenic species isolated from alders in the Iberian Peninsula; Jung et al., 2013; Haque et al., 2015; Ferreira et al., 2022; Horta Jung et al., 2024) suggest that the oomycete with the highest pathogenicity on alder is *P. plurivora* (Zamora-Ballesteros et al., 2017; Corcobado et al., 2023; Vieites-Blanco et al., 2023). This difference in pathogenicity may depend on many factors, such as the alder species and their defense mechanisms, the environmental factors of different riparian forests and the isolates of the *Phytophthora* pathogen tested and their infection capacity. It is important to note that the higher pathogenicity of *P. plurivora* compared to *P. ×aln*i may be due to two factors. First, infection of *P. plurivora* causes a low response in alder, which implies a low defense against this pathogen. Secondly, *P. plurivora* colonizes the xylem and phloem, in contrast to *P. ×alni* which primarily occurs in the phloem. The ability to invade the xylem may provide *P. plurivora* with a competitive advantage over *P.* ×*alni* (Vieites-Blanco et al., 2023). *Phytophthora lacustris* Brasier, Cacciola, Nechwatal, Jung & Bakonyi (**Figure 1B**, **Table 2**, Rial-Martínez et al., 2023) and *P. hydropathica* Hong & Gallegly (**Table 2**, Pintos-Varela et al., 2016) were also detected in alder trees or river water associated with riparian alder, but their pathogenicity remains to be demonstrated.

## Ecological impact of alder disease caused by *Phytophthora* species in riparian and wetland ecosystems

**Figure 4:**
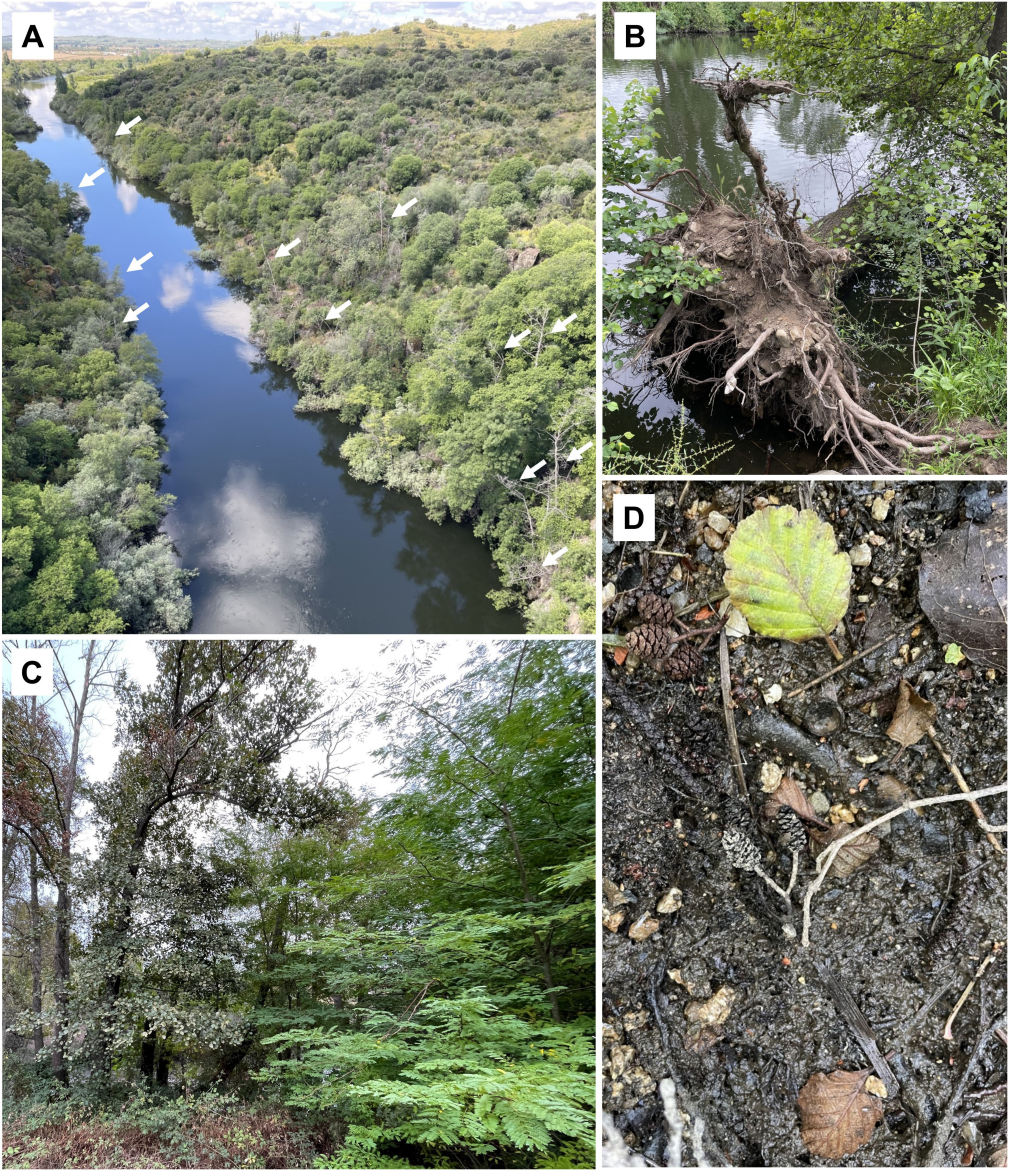
**(A)** Widespread mortality of *Alnus glutinosa* trees (white arrows) induced by *Phytophthora* species along Alagón River (Valdeobispo, Spain). **(B)** Unhealthy alder trees are prone to fall allowing increased soil erosion. **(C)** The gap created by tree mortality due to *P. ×alni* infection will most likely allow rapid colonization of invasive species, such as *Gledistia triacanthos* tree on the left. **(D)** *Phytophthora*-infested soil might not allow seed germination, impeding the successful recruitment of alders; the image shows cones full of viable seeds fallen from alder trees in an infested soil where no regeneration has been registered.

Given the ecological importance of alders (**Table 1**), their disappearance from riparian forests, due to *Phytophthora* occurrence, will likely alter the plant community diversity and structure, and affect the characteristics of streams, because of modified channel morphology, decreased bank stability and increased water temperature (**Figure 4A**). Root rot caused by the pathogen will result in reduced tree stability, and fallen trees in riverbanks will cause accelerated erosion, affecting river geomorphological conditions and increasing damages caused by flooding in crops and farms (**Figure 4B**). Also, with alder death, the loss of woody adventitious roots will reduce the instream habitat available to aquatic organisms, and the loss of wide-canopy trees will reduce the habitat availability to terrestrial organisms. Moreover, the disappearance of alder leaf litter from autumn leaf fall will reduce the supply of high-quality organic matter, disrupting the stream detrital pathway (Alonso et al., 2021). Consequently, instream nutrient cycling will be impaired due to the decreased decomposition rate of leaf litter, caused by the loss of high-quality alder leaf litter (Rubio-Ríos et al., 2023). Additionally, the absence of alder will stop contributing to the increase of the quality of leaf litter from non-nitrogen-fixing species (Rhoades et al., 2001), which may decompose slower due to the loss of the stimulatory effect of alder litter presence on the decomposition of other low-quality leaf litter in mixtures (Ferreira et al., 2012; Alonso et al., 2024).

Even before alder trees completely disappear from streamside forests due to the disease, sick alder trees might already be affecting the functioning of the stream ecosystem. For instance, diseased alder trees generally have sparse and small-sized leaves (Jung et al., 2018), and their leaves have lower nitrogen and higher phosphorus concentrations compared with leaves from healthy trees (Ferreira et al., 2022). As aforementioned, these changes will reduce the amount of leaf litter inputs to streams and likely affect the instream cycling of litter-derived nutrients. Microbial-mediated leaf litter decomposition was faster for Iberian alder trees infected with *P. alni* complex than for healthy trees, probably because of the higher litter phosphorus concentration in diseased trees (Ferreira et al., 2022).

On the other hand, the mortality of alder trees by *Phytophthora* often generates landscape gaps, which trigger germination and proliferation of some exotic invasive species (**Figure 4C**), such as *Acacia dealbata* Link and *A. melanoxylon* R. Br. in specific areas (Portela-Pereira et al., 2022), leading to biodiversity homogenization. Dieback of mother trees induced by *Phytophthora* may hamper forest succession by reducing the rate of successful recruitment events, thus compromising the long-term sustainability of the community (Rodríguez-González et al., 2010). Moreover, soil and water infestation by *Phytophthora* species may impede the successful regeneration of alders, by producing damage to the embryo and radicle of seeds during germination, as reported in other *Phytophthora*-infested ecosystems (Martín-García et al., 2015; **Figure 4D**). Thus, direct mortality along with indirect effects on the alder life cycle might lead to non-linear changes in community composition threatening biodiversity hosted by alder-dominated forests (Biurrun et al., 2021).

## Resistance and tolerance responses against *Phytophthora*

Identifying natural resistant genotypes and understanding the underlying mechanisms of resistance are essential for developing effective management and conservation strategies (Redondo et al., 2020). Resistance and tolerance responses of alders to *Phytophthora* infection are complex and multifaceted, involving a combination of genetic, physiological, biochemical and environmental factors (Gomes Marques et al., 2022; Avila-Quezada and Rai, 2023; Macháčová et al., 2024). Alders have physical barriers as structural defenses, like bark and lignified cell walls (Schmitt et al., 2021). However, *Phytophthora* can penetrate the host through its fine roots and then spread upwards to the trunk, or directly infect the trunk through existing wounds during flooding (Oßwald et al., 2014). This renders the physical barriers of the alder trees ineffective against infection by *Phytophthora*.

Alders have also developed defense mechanisms that define their tolerance or resistance and the level of pathogenicity of the oomycete. For example, trees can use tylose production, lignin deposition and/or callose production and deposition around sieve plates to prevent the infection of vascular systems (Redondo et al., 2015a; van den Berg et al., 2018). On alders, *Phytophthora* was found to affect the epidermis, cortex and vascular cylinder of roots, thus altering fibers and vessels, which leads to a detectable increase in tylose production (Vieites-Blanco et al., 2023). Indeed, some authors relate the presence of tyloses to a defense mechanism to hamper the advance of mycelium (Narayan et al., 2022; Martín and López, 2023). However, Vieites-Blanco et al. (2023) considered that the production of tyloses could be an indicator of damage rather than of plant resistance. In turn, callose formation in the crib plates is associated with plant resistance to *Phytophthora*. Thus, van den Berg et al. (2018) observed a lack of callose formation on susceptible roots with invasive hyphae, and Vieites-Blanco et al. (2023) detected differences in callose formation in alders infected with less aggressive pathogens vs. absence of callose with the more aggressive pathogens.

A direct relationship has been observed between the geographical distribution of *Phytophthora* species and subspecies and the climatic conditions. Efforts to create a comprehensive and reliable map of the global spread of various *Phytophthora* species have revealed that their distribution is related with the abiotic conditions of the studied areas. For example, in southern Sweden, a temperature-related barrier separates the survival of two species: *P. uniformis*, which can withstand lower temperatures, and *P. ×alni* which is only found in southern regions with milder climatic conditions (Redondo et al., 2015b, Redondo et al., 2020; Teshome et al., 2020).

In addition, biological barriers, such as microbial communities, are very important in enhancing plant defenses. Forest trees maintain close relationships with a wide variety of microorganisms that are essential for maintaining tree health, optimizing nutrient availability and supporting overall ecosystem functions (Fuller et al., 2023). Compared to crops planted yearly, the microbes that live around trees, both helpful and harmful, usually have more steady and stable interactions. This stability is largely due to the deep root systems of trees, which create a more stable environment for microbial communities (Mercado-Blanco et al., 2018). Beneficial microbiota, including plant growth-promoting rhizobacteria (PGPR), plant growth-promoting fungi (PGPF) and biocontrol agents, can significantly influence the metabolic processes of alder trees. These microorganisms enhance growth, improve performance and increase the trees’ resistance to various stresses in an economically efficient manner (Tian et al., 2020). Furthermore, in the search for resistant or tolerant genotypes to the pathogen *Phytophthora*, it was observed that progenies of alders from an area invaded by *P. uniformis* were less susceptible to *P. ×alni* than progenies from a pathogen-free zone. This could suggest an epigenetic regulation in some of the mechanisms of alder resistance to *Phytophthora*, as recently described in citrus (Rodrigues da Silva et al., 2021). Also, these studies provide valuable information, as they confirm that responses to infection can be inherited and highlight the importance of studying both surviving and healthy trees (not just the diseased ones), since they provide adaptation potential to the future generation. However, they also stress the need to study the stability of these responses over time and generations.

Through research, several potential biological control agents (BCAs) have been identified to reduce pathogenic spread and associated symptoms including dieback and root rot. The most commonly used BCAs belong to the genera *Pseudomonas, Bacillus* and *Trichoderma.* For instance, a study by Zaspel et al. (2014) demonstrated the advantages of root treatment with *Pseudomonas veronii* Coroler, Elomari, Hoste, Gillis, Izard, Leclerc for enhancing alder rooting. The study found that this bacterium can induce tolerance in some *P. ×alni*-infected alders, allowing them to survive without exhibiting symptoms caused by the pathogen. Moreover, while studies of BCAs on alder are limited, there are many registered microorganisms with antagonistic activity on other *Phytophthora* pathogens whose benefits have been successfully demonstrated. For example, *Pseudomonas putida* (Trevisan) Migula and *P. chlororaphis* (Guignard and Sauvageau, 1894; Bergey et al., 1930) were used as BCA of *Phytophthora* root rot in citrus orchids (Steddom et al., 2002) and in *P. palmivora* E.J. Butler infected-cacao plants, respectively (Acebo-Guerrero et al., 2015). In the same way, *Bacillus amyloliquefaciens* (Fukomoto) Priest, Goodfellow, Shute & Berkeley induced systemic resistance against *P. cactorum* (Lebert & Cohn) J. Schroüt (Lee et al., 2015). In addition, *Trichoderma virens* (J.H. Miller, Giddens & A.A. Foster) Arx, *T. harzianum* Rifai, *T. asperellum* Samuels, Lieckfeldt & Nirenberg and *T. spirale* Bissett showed antagonistic effects against *P. palmivora* in cacao plants (Mpika et al., 2009). *T. saturnisporum* Hammill also showed antagonistic effects against several *Phytophthora* spp (Diánez Martínez et al., 2016; Mercado-Blanco et al., 2018). These findings suggest a promising avenue for biocontrol against *Phytophthora* species, underscoring the importance of investigating the alder microbiome. Such research aims to identify potential microorganisms capable of inhibiting or parasitizing the pathogen decreasing its pathogenicity, thereby enhancing the host chances of survival and resilience (Redondo et al., 2020).

## Breeding programs and approaches to fight against diseases and pests in forest tree species that may guide research on alder

Forest tree breeding is a laborious and time-consuming process, strongly limited by the long breeding cycle of most tree species. An important factor in maintaining the long-term viability of the alder populations in Europe is the development or maintenance of natural resistance to *Phytophthora*. Additionally, the presence of asymptomatic alders surviving in highly affected areas could suggest the potential resistance to alder dieback in some common alder genotypes (Jung and Blaschke, 2004). Traditional approaches for breeding are based on the selection and mating of elite trees carrying the desirable traits. It is crucial to identify asymptomatic genotypes, which may be resistant, within natural populations affected by the pathogen. These genotypes should be tested for resistance under controlled conditions, typically using a combination of short- and long-term assessments. This includes planting the selected genotypes in areas with high disease pressure and varying environmental conditions (Sniezko, 2006; Sniezko and Koch, 2017). The confirmed resistant trees can then be used to develop a pool of resistant germplasm, for reforestation or restoration purposes, by clonal propagation and/or by crossing with other resistant trees (Keriö et al., 2019).

Forest biotechnology provides tools for conserving and sustainably managing of natural genetic resources, as well as for optimizing and speeding up genetic improvement programs. The development and use of biotechnological techniques provide, on the one hand, information for better management of natural resources, through the study of the genetic variability of forest tree resources and their functional characterization, employing molecular biology techniques (Díaz-Sala, 2014, 2019). On the other hand, *in vitro* culture techniques make possible large-scale plant propagation within breeding programs to, among others, preserve selected outstanding genotypes that are difficult to conserve by other methods. The application of tissue culture techniques has also led to the development of cryopreservation, as an additional strategy for conservation widely used in the agricultural sector. Vegetative propagation and *in vitro* propagation allow the development of clonal trials for phenotypic evaluation, the multiplication and maintenance of the genotypes of interest, the regeneration of a high number of plants in breeding programs and guarantee the health and availability of forest material quickly at any time and season of the year (Díaz-Sala, 2016).

### Large-scale vegetative propagation

Alders are easily propagated by seeds, and this is the most used method when a high number of specimens is required. However, due to the variation resulting from sexual reproduction, the use of seeds as a means of propagation to produce plants expressing a desirable trait, such as disease resistance, is limited, and vegetative propagation methods would be more appropriate. An alternative to sexual reproduction, for capturing genetic gains, is the vegetative propagation of trees that show desirable traits. Vegetative propagation also allows the preservation of non-additive gene effects, which result from gene interactions. These effects are usually not passed on through sexual reproduction but can produce exceptional individuals. In horticulture, mass vegetative propagation of selected phenotypes has been used for centuries. However, woody species have specific characteristics that make mass propagation not widely used in forestry, despite the need to propagate elite genotypes by these methods (Greenwood and Weir, 1995). The high heterozygosity of forest species, combined with the significant non-additive genetic effects influencing various traits of interest, requires the use of vegetative propagation to achieve optimal genetic gains while preserving the genotype’s identity and biodiversity.

Alders can be propagated vegetatively by rooting woody cuttings. However, rooting success is highly dependent on the genotype, tree age, collection season, type of cuttings and treatments used. Even though, annual softwood cuttings were found more appropriate for rooting and vegetative propagation of mature common alder trees (Radwan et al., 1989; Novotná and Štochlová, 2012). Also, cuttings collected during the breakdown of endogenous dormancy of the mother plant (December to February), or before the onset of dormancy (July to September), seem to be more successful for rooting of *A. glutinosa* (Novotná and Štochlová, 2012).

Alders can also be propagated by using *in vitro* tissue culture techniques (Tremblay and Lalonde, 1984; Périnet and Tremblay, 1987; Corredoira et al., 2011, Corredoira et al., 2013; Bajji et al., 2013; San José et al., 2013). Indeed, *in vitro* propagation and conservation of alders have been carried out by somatic embryogenesis and subsequent cryopreservation of induced somatic embryos (Corredoira et al., 2013; San José et al., 2015b). However, at present, the establishment of axillary micropropagation systems, by sequential subcultures and subsequent rooting of the shoots obtained, is the most widely used method, as it guarantees a rapid large-scale multiplication and genetic stability (Tremblay and Lalonde, 1984; Périnet and Tremblay, 1987; Corredoira et al., 2011; San José et al., 2012, San José et al., 2013). However, the effect of the genotype, the tree age and phytosanitary conditions, the stage of development and the status of the explant material are major factors affecting culture establishment (Tremblay and Lalonde, 1984; Périnet and Tremblay, 1987; Bajji et al., 2013; San José et al., 2013). Remarkably, the use of explants from forced-to-flush axillary shoots, from mature branches under controlled conditions, avoids the huge contamination problems when explants taken directly from the field are used for the initiation of *in vitro* cultures (Corredoira et al., 2011; San José et al., 2013). A specific culture medium for woody species, supplemented with cytokinins and auxins, is required for the successful establishment and multiplication of juvenile and adult tree explants by shoot cultures (Bajji et al., 2013; San José et al., 2013). In addition, the carbohydrate source seems to play an important role in both multiplication and rooting phases of shoots (San José et al., 2011, San José et al., 2013). In the presence of exogenous auxin, primarily indole-3-butyric acid, or even in its absence, alder induces adventitious roots in stems developed *in vitro* (Corredoira et al., 2011; San José et al., 2012, San José et al., 2013). Recently, the use of *in vitro* systems of temporary immersion in a liquid medium has been described as an alternative strategy for the improvement of alder multiplication (San José et al., 2020). In the same way, the use of double-phase culture systems, by adding liquid medium (Rodriguez et al., 1991), improves the multiplication rates and plant vigor, while reducing the management costs. The storage of *A. glutinosa* shoot cultures under minimum growth conditions allows maintaining cultures for extended periods (up to 18 months before subculturing), which results in a cost-efficient storage of alder germplasm, thus contributing to improved conservation of alder genetic diversity (San José et al., 2015a).

### Recent advances to improve the efficiency of tree breeding

Although forest tree breeding usually lasts for decades, recent advancements in methods and strategies have introduced tools to accelerate and refine this process. Genomic Selection (GS), Marker-Assisted Selection (MAS), Genome-Wide Association Studies (GWAS) and Quantitative Trait Loci (QTL) have revolutionized tree breeding by enabling the precise identification and manipulation of genes associated with resistance to pests and diseases (Fan et al., 2024; Fernandes et al., 2024; Jacobs et al., 2024; Sharma et al., 2024). These approaches have great potential to augment and help advance tree improvement programs, through early, indirect selection of improved genotypes (Sniezko and Koch, 2017). Moreover, technologies such as the CRISPR/Cas9 genome-editing tool allow for precise alterations in tree genomes, opening the door for obtaining individuals with specific resistances to pests and diseases. This technology, combined with GS and MAS, could help to accelerate breeding cycles and improve the genetic gain per generation (Poovaiah et al., 2021). In addition, modern High-Throughput Phenotyping (HTP) technologies, like cameras, sensors, Unmanned Aerial Vehicles (UAV), robotics and computers, allow the collection of reliable phenotypic data of thousands of individuals with unprecedented speed and accuracy, *i.e.* automated phenotyping (aka phenomics) (Spalding and Miller, 2013). For instance, these technologies (*e.g.* UAV) might be used to identify volatiles or any other chemical signal linked with resistant phenotypes (Quintana-Rodríguez et al., 2015) and/or with the maturity stage of the pest (Mamidala et al., 2013; Shen et al., 2021). For instance, UAVs were used to monitor the health status of trees affected by alder dieback in Northern Portugal (Guerra-Hernández et al., 2021). Recent advances in Artificial Intelligence may also leverage the linking of phenotypes to genomic features (Rairdin et al., 2022), which is particularly challenging in the current big data era in plant biology (Deng et al., 2023), as well as the phenotype/disease identification/diagnosis (Ferentinos, 2018; Wang et al., 2022) and the GS prediction models (Sharma et al., 2024).

### Case studies in breeding against forest pests and diseases

The Emerald Ash Borer (EAB, *Agrilus planipennis* Fairmaire) case, a prime example of an invasive pest introduced through globalization, is an example of how multiple biotechnological approaches to genetic breeding can help in disease control in forest species. Native to Northeast Asia, EAB was first found in the USA in 2002 and, since then, it has devastated ash populations (*Fraxinus* spp.), including green ash (*F. pennsylvanica* Marsh), and forested ecosystems, with severe economic and ecological impacts (Herms and McCullough, 2014). Thus, EAB is the most damaging invasive forest insect pest ever to have invaded North America, threatening nearly all native species of *Fraxinus* with functional extinction (Poland and McCullough, 2006; Herms and McCullough, 2014; Aubin et al., 2015; Stanley et al., 2023). Breeding programs have focused on identifying and propagating EAB-resistant ash trees. Research has identified specific genetic markers associated with resistance, enabling the use of MAS to accelerate breeding efforts (Cobo-Simón et al., 2021; Huff et al., 2022). In addition, ongoing genomic studies aim to understand the mechanisms of resistance and enhance the resilience of ash populations (Huff et al., 2022; Battisti and Larsson, 2023). However, further knowledge of the ash genome is of vital importance to understanding the genetic basis of ash resistance. In this sense, the recently published genomes of *F. excelsior* L. and *F. pennsylvanica* can be very useful (Sollars et al., 2017; Huff et al., 2022). Another approach has optimized the *Agrobacterium*-mediated transformation system to introduce a Bt toxin gene into transgenic *F. nigra* Marshall susceptible shoots (Lee and Pijut, 2018). From another perspective, Merkle et al. (2023) reported the application of somatic embryogenesis to clonally propagate progeny of lingering *F. americana* L. and *F. pennsylvanica* parents, which could provide EAB-resistant ash varieties for forest and urban tree restoration. Moreover, transcriptome and proteomic analyses also provide information and powerful tools to advance pedigree-based breeding and selection programs as well as the management of standing populations (Neale and Kremer, 2011). Recently, Chiu et al. (2023) identified a unique set of genes linked to three different levels of increasing EAB infestation of *F. pennsylvanica*. Moreover, studies on resistance variability and candidate genes for ash tree stress defense can help breeding for resistance to EAB (Lane et al., 2016; Kelly et al., 2020). Although there is still much progress to be made in the fight against EAB, the combined use of several biotechnological approaches could offer new hope for the survival of North American *Fraxinus* species.

Several other case studies highlight the success of modern forest breeding programs. For instance, breeding programs for Norway spruce (*Picea abies* [L.] H. Karst) and various pine species (*Pinus* spp.) have shown significant improvements in growth and resistance traits using GS techniques (Sharma et al., 2024). These programs use genetic data to predict and select the best candidates for breeding, enhancing resistance to common pests and diseases such as bark beetles (Westbrook et al., 2013), whose impacts are magnified by climate change. These efforts have resulted in more resilient tree populations capable of withstanding pest invasions and environmental stresses (Lenz et al., 2020; Sharma et al., 2024).

American chestnut (*Castanea dentata* (Marshall) Borkh.) was nearly eradicated by chestnut blight (*Cryphonectria parasitica* (Murrill) Barr). Breeding programs have aimed to restore this iconic species through hybridization with blight-resistant Chinese chestnut (*C. mollissima* Blume). American chestnut is also highly susceptible to the soil-borne pathogen *P. cinnamomi* Rands, which causes root rot. This pathogen is spreading towards northern regions of North America due to climate change. Breeding programs aim to combine resistance to chestnut blight and *Phytophthora*, by breeding blight-resistant hybrids with *P. cinnamon*-resistant American chestnuts. Recent advances in GS,CRISPR/Cas9 technology (Westbrook et al., 2019a, Westbrook et al., 2019b; Fernandes et al., 2022; Battisti and Larsson, 2023) and transcriptomics, particularly comparative transcriptome analysis (Barakat et al., 2009, Barakat et al., 2012; Nie et al., 2023), have furthered these efforts, leading to crucial findings to assist successful breeding and offering hope for the re-establishment of American chestnut populations.

In European chestnut species, breeding for resistance to ink disease, caused by *P. cinnamomi*, allowed for the development of tolerant hybrid rootstocks in Europe, by crossing the local *C. sativa* Mill. with the two Asian tolerant species*, C. crenata* Sieb. and Zucc. and *C. mollissima* (Miranda-Fontaíña et al., 2007; Santos et al., 2015, Santos et al., 2017). Furthermore, the selection of *P. cinnamomi*-tolerant chestnut trees has been evaluated using EST-SSRs, among which the CsPT_0005 locus could be applied in marker-assisted selection to predict *P. cinnamomi* resistance in non-inoculated *C. sativa* trees (Alcaide et al., 2020).

In Spain, a breeding program is currently underway to obtain *Quercus ilex* L. and *Q. suber* L. varieties tolerant to *P. cinnamomi*. It includes activities such as the identification of trees in affected areas and selection of symptomless individuals, population variability studies, propagation of symptomless specimens identified in affected areas, evaluation of the suitability of tolerant material as rootstocks, tolerance trials “*in vitro*”, in the nursery and the field, installation of seed orchards of selected clonal materials, search for molecular markers linked to resistance/tolerance responses, and studies of the biological component of the soil (Tapias et al., 2006, Tapias et al., 2008; Pérez et al., 2020; Martínez et al., 2023).

Genetic improvement has also proven to be effective as a control tool against the pine wood nematode (*Bursaphelenchus xylophilus* (Steiner and Buhrer) Nickle). Designed to combat this pathogen, the genetic breeding program from the Forestry Research Center of Lourizán (Spain) has successfully developed resistant varieties of *Pinus pinaster* Aiton, that are now offered for sale in nurseries (Díaz-Vázquez et al., 2020). For this purpose, researchers studied variation in susceptibility to the nematode across several pine species, as well as among *P. pinaster* provenances from the Iberian Peninsula and France, and among half-sib families within the *P. pinaster* and *P. radiata* D. Don genetic improvement programs (Díaz-Vázquez et al., 2020; Menéndez-Gutiérrez et al., 2018, Menéndez-Gutiérrez et al., 2021).

The breeding program for *Chamaecyparis lawsoniana* (A. Murray bis) Parl. (Port-Orford cedar or Lawson’s cypress) resistance to *P. lateralis* Tucker & Milbrath, in the USA, is one of the most promising resistance selection efforts for forest trees around the world (Keriö et al., 2019). *P. lateralis* nearly decimated *C. lawsoniana*, a keystone tree species in the Pacific Northwest and one of the most valued landscape tree species in the Northern Hemisphere. Currently, containerized seed orchards with the best parents and progeny are maintained for resistance testing. Seed production and field plantings indicate high survival rates of the resistant planting stock (Hansen et al., 2012).

Unfortunately, there are relatively few examples of tree breeding programs focused on *Phytophthora* resistance in wild forest species, compared to those with higher economic significance, such as crops. In contrast, agricultural species have benefited from genomic techniques such as QTL mapping, GWAS, and genome-wide extreme phenotyping (XP-GWAS). These approaches have been instrumental in identifying significant QTLs and single nucleotide polymorphisms (SNPs) associated with resistance to *Phytophthora* (Siviero et al., 2006; Siddique et al., 2019; de Ronne et al., 2022; Ro et al., 2022; Li et al., 2023; Lin et al., 2023). For example, underway breeding programs have allowed obtaining citrus rootstocks resistant to *P. nicotianae* Breda de Hann, and the use of molecular technologies, linkage maps and QTL information has improved the efficiency of various citrus breeding programs, by decreasing evaluation times for a high number of genotypes and providing study targets (Lima et al., 2018).

Since the disease caused by *Phytophthora* was first detected in European alders in the 1990s, it has resulted in severe losses in timber production, and severe damage to alder stands in forests and along riverbanks with negative effects on flood defense and biodiversity loss. Forest management strategies and fungicides are the usual way of coping with this disease, but the control of the pathogen with chemical treatments outside nurseries is not allowed. Therefore, breeding for resistance can be one of the most effective strategies to control the disease. Furthermore, the need to preserve genetic diversity and the presence of tolerant alders support the need for breeding for resistance. However, until recently, the only known breeding program for common alder resistant to *P. ×alni* is the RESISTANT ALDER project, conducted at the Institute of Forest Genetics in Waldsieversdorf (Germany). This project ended in 2017, resulting in the selection of only four clones with lower susceptibility to *P. ×alni*.

**Figure 5:**
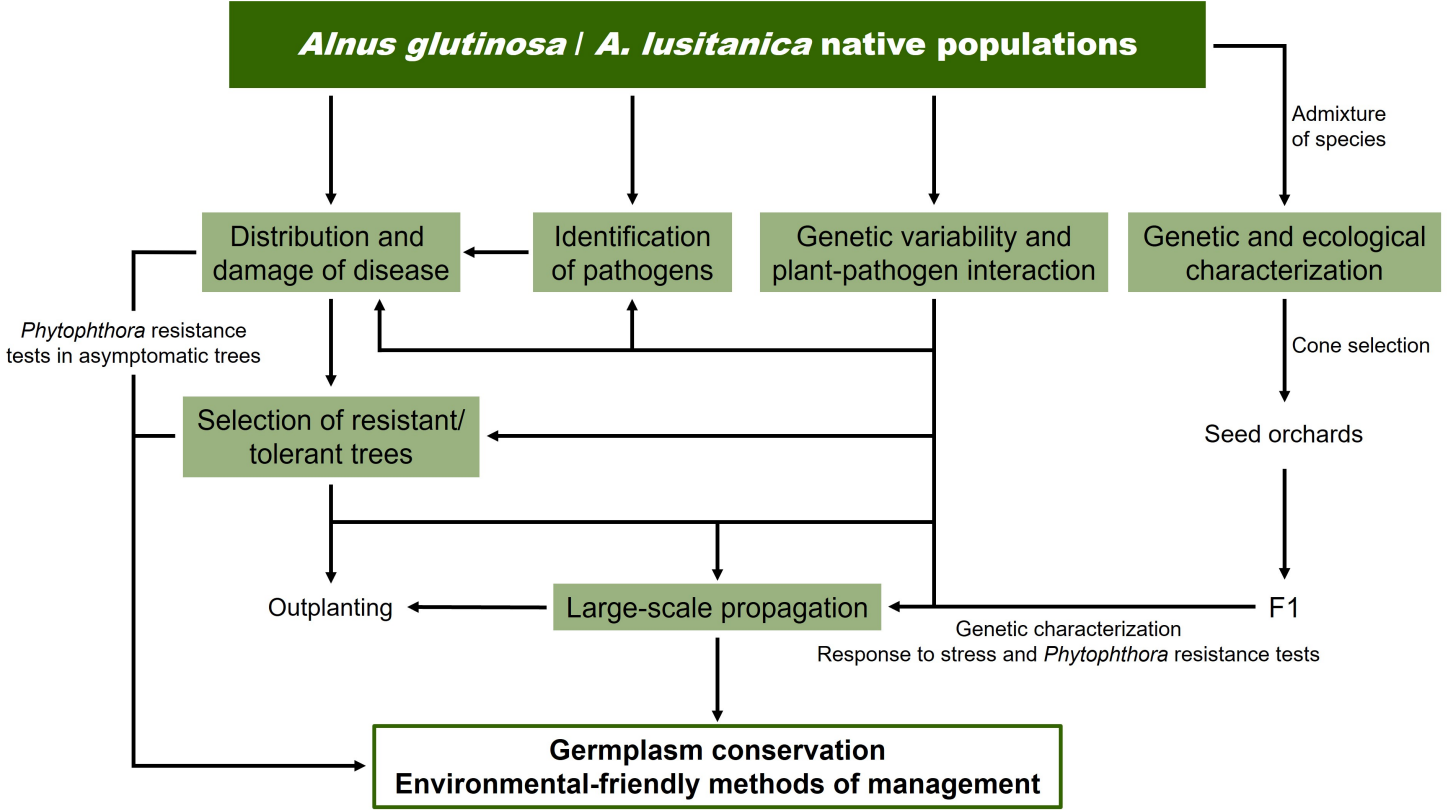
Program launched in RETAIN and ATLANTES projects to tackle alder decline in Spanish riparian forest ecosystems. Information on the program launched in the Portuguese ALNUS project can be found at https://www.isa.ulisboa.pt/proj/alnus/project/.

In response to the widespread alder mortality in the Iberian Peninsula, comprehensive programs have recently been launched to address the alder decline in Spanish and Portuguese riparian forest ecosystems. The ALNUS project in Portugal (2018-2022; https://www.isa.ulisboa.pt/proj/alnus/project/) launched the study of alder populations’ resilience to *Phytophthora* and climate change. In Spain, the RETAIN (2022-2024) and ATLANTES (2022-2025) projects, involving several research groups from different universities and research institutes, with the collaboration of public bodies in charge of alder management (Ministry for Ecological Transition and the Demographic Challenge, River Basin Authority and Regional Government of Castile-La Mancha), initiated an ambitious regional and national program to cope with alder decline in Spanish riparian forest ecosystems (**Figure 5**). This program involves, on one side, the determination of the current distribution and damage of alder dieback, as well as the characterization of the *Phytophthora* species complex causing the disease, to improve our understanding of host-pathogen interactions. On the other side, it plans to carry out an in-depth characterization of the existing genetic variability of alders from the Iberian Peninsula and the interactions in the *Alnus-Phytophthora* pathosystem, along with the identification and large-scale propagation of resistant or less susceptible trees while maintaining biodiversity. The genetic and ecological characterization of an admixture of species in an *Alnus* stand, as well as of their offspring in response to abiotic and biotic factors are also included in the program. This information will allow, when planning a restoration program, to consider any genetic structure at a local scale and differentiation at a river catchment (Rodríguez-González et al., 2019), provenance region or national scale. Maintaining genetic diversity within forest populations during breeding for resistance is crucial to ensure resilience against future threats (Blows and Hoffmann, 2005; Bijlsma and Loeschcke, 2012; Hamilton et al., 2017; Sjöman et al., 2024). Alimpić et al. (2022) concluded that a combination of *in situ* and *ex situ* measures and/or integrative conservation of riparian ecosystems is the most appropriate option for conserving the genetic diversity of riparian tree species. In addition, in the Iberian Peninsula, where two species (*A. glutinosa* and *A. lusitanica*), hard to distinguish, coexist, traceability may be of particular interest to river managers.

## Conclusions and future perspective

Alders play significant ecological, commercial and recreational roles. However, a strong reduction of alder stands has been detected in Europe due to infection by *Phytophthora* spp. Continuous tree germplasm screening, effective procedures for the evaluation of the disease, accurate identification of *Phytophthora* species and identification of tolerant genotypes for large-scale propagation are crucial for the development of breeding programs that will preserve tree genetic diversity. The advances in genetic screening and genomic technologies, reference genomes and bioinformatic tools, including the promising application of Artificial Intelligence techniques in molecular biology (e.g. AlphaFold; Tunyasuvunakool et al., 2021; Abramson et al., 2024), as well as the genomics of *Phytophtora*-alder interactions and the novel high-throughput phenotyping techniques, will help to identify resistance gene(s), QTLs and pathogen effectors. This knowledge will allow the application of genomic-assisted breeding, gene silencing and gene editing to improve *Phytophthora* resistance in alder.

## References

[B1] Abramson J. Adler J. Dunger J. Evans R. Green T. Pritzel A.. (2024). Accurate structure prediction of biomolecular interactions with AlphaFold 3. Nature 630, 493–500. doi: 10.1038/s41586-024-07487-w 38718835 PMC11168924

[B2] Acebo-Guerrero Y. Hernández-Rodríguez A. Vandeputte O. Miguélez-Sierra Y. Heydrich-Pérez M. Ye L.. (2015). Characterization of *Pseudomonas chlororaphis* from *Theobroma cacao* L. rhizosphere with antagonistic activity against *Phytophthora palmivora* (Butler). J. Appl. Microbiol. 119, 1112–1126. doi: 10.1111/jam.12910 26218193

[B3] Alcaide F. Solla A. Cherubini M. Mattioni C. Cuenca B. Camisón Á.. (2020). Adaptive evolution of chestnut forests to the impact of ink disease in Spain. J. Syst. Evol. 58, 504–516. doi: 10.1111/jse.12551

[B4] Alimpić F. Milovanović J. Pielech R. Hinkov G. Jansson R. Dufour S.. (2022). The status and role of genetic diversity of trees for the conservation and management of riparian ecosystems: a European experts' perspective. J. Appl. Ecol. 59, 2476–2485. doi: 10.1111/1365-2664.14247

[B5] Alonso A. Boyero L. Solla A. Ferreira V. (2024). Dieback and replacement of riparian trees may impact stream ecosystem functioning. Microb. Ecol. 87, 32. doi: 10.1007/s00248-024-02343-w 38228918 PMC10791780

[B6] Alonso A. Pérez J. Monroy S. Lopez-Rojo N. Basaguren A. Bosch J.. (2021). Loss of key riparian plant species impacts stream ecosystem functioning. Ecosystems 24, 1436–1449. doi: 10.1007/s10021-020-00592-7

[B7] Aubin I. Cardou F. Ryall K. Kreutzweiser D. Scarr T. (2015). Ash regeneration capacity after emerald ash borer (Eab) outbreaks: some early results. For. Chron. 91, 291–298. doi: 10.5558/tfc2015-050

[B8] Avila-Quezada G. D. Rai M. (2023). Novel nanotechnological approaches for managing *Phytophthora* diseases of plants. Trends Plant Sci. 28, 1070–1080. doi: 10.1016/j.tplants.2023.03.022 37085411

[B9] Bajji M. Thunissen C. Druart P. (2013). Adventitious shoot regeneration from in *vitro* juvenile explants of black alder (*Alnus glutinosa* [L.] Gaertn.). Biotechnol. Agron. Soc Environ. 17, 12–19.

[B10] Barakat A. DiLoreto D. S. Zhang Y. Smith C. Baier K. Powell W. A.. (2009). Comparison of the transcriptomes of American chestnut (*Castanea dentata*) and Chinese chestnut (*Castanea mollissima*) in response to the chestnut blight infection. BMC Plant Biol. 9, 51. doi: 10.1186/1471-2229-9-51 19426529 PMC2688492

[B11] Barakat A. Staton M. Cheng C. H. Park J. Yassin N. B. M. Ficklin S.. (2012). Chestnut resistance to the blight disease: insights from transcriptome analysis. BMC Plant Biol. 12, 38. doi: 10.1186/1471-2229-12-38 22429310 PMC3376029

[B12] Battisti A. Larsson S. (2023). “Climate change and forest insect pests,” in Forest Entomology and Pathology. Eds. Allison J. D. Slippers B. Wingfield M. J. (Springer, Cham, Switzerland), 773–787. doi: 10.1007/978-3-031-11553-0_22

[B13] Beatty G. Montgomery W. Tosh D. Provan J. (2015). Genetic provenance and best practice woodland management: a case study in native alder (*Alnus glutinosa*). Tree Genet. Genomes 11, 92. doi: 10.1007/s11295-015-0919-1

[B14] Bergey D. H. Harrison F. C. Breed R. S. Hammer B. W. Huntoon F. M. (1930). Bergey's manual of determinative bacteriology. 3rd ed (Baltimore: The Williams and Wilkins Co.).

[B15] Bijlsma R. Loeschcke V. (2012). Genetic erosion impedes adaptive responses to stressful environments. Evol. Appl. 5, 117–129. doi: 10.1111/j.1752-4571.2011.00214.x 25568035 PMC3353342

[B16] Biurrun I. Portela-Pereira E. Mendías C. S. Rodríguez-González P. M. (2021). “ *Osmundo*-*Alnion glutinosae* (Br.-Bl. et al. 1956) Dierschke et Rivas-Mart. in Rivas-Mart. 1975: Floodplain forests of the western (Atlantic half of the Iberian Peninsula,” in Interpretative manual of European riparian forests and shrublands. Eds. Mandžukovski D. Čarni A. Sotirovski K. (Ss Cyril and Methodius University, Skopje, North Macedonia), 21–26.

[B17] Bjelke U. Boberg J. Oliva J. Tattersdill K. McKie B. G. (2016). Dieback of riparian alder caused by the *Phytophthora alni* complex: projected consequences for stream ecosystems. Freshw. Biol. 61, 565–579. doi: 10.1111/fwb.12729

[B18] Blows M. W. Hoffmann A. A. (2005). A reassessment of genetic limits to evolutionary change. Ecology 86, 1371–1384. doi: 10.1890/04-1209

[B19] Brasier C. M. Kirk S. A. (2001). Comparative aggressiveness of standard and variant hybrid alder Phytophthoras, *Phytophthora cambivora* and other *Phytophthor*a species on bark of *Alnus*, *Quercus* and other woody hosts. Plant Pathol. 50, 218–229. doi: 10.1046/j.1365-3059.2001.00553.x

[B20] Brasier C. M. Rose J. Gibbs J. N. (1995). An unusual *Phytophthora* associated with widespread alder mortality in Britain. Plant Pathol. 44, 999–1007. doi: 10.1111/j.1365-3059.1995.tb02658.x

[B21] Brasier C. Scanu B. Cooke D. Jung T. (2022). *Phytophthora*: an ancient, historic, biologically and structurally cohesive and evolutionarily successful generic concept in need of preservation. IMA Fungus 13, 12. doi: 10.1186/s43008-022-00097-z 35761420 PMC9235178

[B22] Bregant C. Batista E. Hilário S. Linaldeddu B. T. Alves A. (2023). *Phytophthora* species involved in *Alnus glutinosa* decline in Portugal. Pathogens 12, 276. doi: 10.3390/pathogens12020276 36839548 PMC9966130

[B23] Černý K. Strnadová V. (2012). Winter survival of *Phytophthora alni* subsp. *alni* in aerial tissues of black alder. J. For. Sci. 58, 328–336. doi: 10.17221/11/2012-JFS

[B24] Chandelier A. Husson C. Druart P. Maçais B. (2016). Assessment of inoculation methods for screening black alder resistance to *Phyophthora xalni* . Plant Pathol. 65, 441–450. doi: 10.1111/ppa.12418

[B25] Chen Q. Bakhshi M. Balci Y. Broders K. D. Cheewangkoon R. Chen S. F.. (2022). Genera of phytopathogenic fungi: GOPHY 4. Stud. Mycol. 101, 417–564. doi: 10.3114/sim.2022.101.06 36059898 PMC9365048

[B26] Chiu C. C. Pelletier G. Stival Sena J. Roux-Dalvai F. Prunier J. Droit A.. (2023). Integrative analysis of green ash phloem transcripts and proteins during an emerald ash borer infestation. BMC Plant Biol. 23, 123. doi: 10.1186/s12870-023-04108-y 36869316 PMC9983263

[B27] Claessens H. Oosterbaan A. Savill P. Rondeux J. (2010). A review of the characteristics of black alder (*Alnus glutinosa* (L.) Gaertn.) and their implications for silvicultural practices. Forestry 83, 163–175. doi: 10.1093/forestry/cpp038

[B28] Cobo-Simón I. Bennett J. Fritz A. Romero-Severson J. Reid M. Koch J.. (2021). “Breeding resistant ash for North America: identifying genetic signatures associated with the lingering phenotype in green ash (*Fraxinus pennsylvanica*),” in Botany 2021 Conference, Virtual. Available at: https://2021.botanyconference.org/engine/search/index.php?func=detail&aid=483.

[B29] Comes H. P. Kadereit J. W. (1998). The effect of Quaternary climatic changes on plant distribution and evolution. Trends Plant Sci. 3, 432–438. doi: 10.1016/S1360-1385(98)0127-2

[B30] Compton J. E. Church M. R. Larned S. T. Hogsett W. E. (2003). Nitrogen export from forested watersheds in the Oregon Coast Range: the role of N_2_-fixing red alder. Ecosystems 6, 773–785. doi: 10.1007/s10021-002-0207-4

[B31] Corcobado T. Cech T. L. Daxer A. Ďatková H. Janoušek J. Patra S.. (2023). *Phytophthora*, *Nothophytophthora* and *Halophytophthora* diversity in rivers, streams and riparian alder ecosystems of Central Europe. Mycol. Prog. 22, 50. doi: 10.1007/s11557-023-01898-1 37323627 PMC10264269

[B32] Corredoira E. Janeiro L. V. San José M. C. (2011). Aplicación de técnicas de cultivo in *vitro* en la propagación del aliso con vistas a su conservación. Recursos Rurais 7, 49–57. doi: 10.15304/rr.id92

[B33] Corredoira E. Valladares S. Martínez M. T. Vieitez A. M. San José M. C. (2013). Somatic embryogenesis in *Alnus glutinosa* (L.) Gaertn. Trees 27, 1597–1608. doi: 10.1007/s00468-013-0907-8

[B34] Cubry P. Gallagher E. O’Connor E. Kelleher C. (2015). Phylogeography and population genetics of black alder (*Alnus glutinosa* (L.) Gaertn.) in Ireland: putting it in a European context. Tree Genet. Genomes 11, 99. doi: 10.1007/s11295-015-0924-4

[B35] De Kort H. Mergeay J. Vander Mijnsbrugge K. Decocq G. Maccherini S. Kehlet Bruun H. H.. (2014b). An evaluation of seed zone delineation using phenotypic and population genomic data on black alder *Alnus glutinosa.* J. Appl. Ecol. 51, 1218–1227. doi: 10.1111/1365-2664.12305

[B36] De Kort H. Vandepitte K. Bruun H. H. Closset-Kopp D. Honnay O. Mergeay J. (2014a). Landscape genomics and a common garden trial reveal adaptive differentiation to temperature across Europe in the tree species *Alnus glutinosa.* Mol. Ecol. 23, 4709–4721. doi: 10.1111/mec.12813 24860941

[B37] Deng C. H. Naithani S. Kumari S. Cobo-Simón I. Quezada-Rodríguez E. H. Skrabisova M.. (2023). Genotype and phenotype data standardization, utilization and integration in the big data era for agricultural sciences. Database, 2023, baad088. doi: 10.1093/database/baad088 38079567 PMC10712715

[B38] de Ronne M. Santhanam P. Cinget B. Labbé C. Lebreton A. Ye H.. (2022). Mapping of partial resistance to *Phytophthora sojae* in soybean PIs using whole-genome sequencing reveals a major QTL. Plant Genome 15, e20184. doi: 10.1002/tpg2.20184 34964282 PMC12807403

[B39] Diánez Martínez F. Santos M. Carretero F. Marín F. (2016). *Trichoderma saturnisporum*, a new biological control agent. J. Sci. Food Agric. 96, 1934–1944. doi: 10.1002/jsfa.7301 26059112

[B40] Díaz-Sala C. (2014). Direct reprogramming of adult somatic cells toward adventitious root formation in forest tree species: the effect of the juvenile-adult transition. Front. Plant Sci. 5. doi: 10.3389/fpls.2014.00310 PMC4083218 25071793

[B41] Díaz-Sala C. (2016). “Physiological, cellular, molecular and genomic analysis of the effect of maturation on propagation capacity,” in Vegetative Propagation of Forest Trees. Eds. Park Y. S. Bonga J. M. Moon H. K. (National Institute of Forest Science, Seoul), 75–96.

[B42] Díaz-Sala C. (2019). Molecular dissection of the regenerative capacity of forest tree species: special focus on conifers. Front. Plant Sci. 9. doi: 10.3389/fpls.2018.01943 PMC6333695 30687348

[B43] Díaz-Vázquez R. Menéndez M. Prada E. (2020). La mejora genética como herramienta de control frente al nematodo del pino. Primeros pinos tolerantes catalogados en España. Foresta 78, 84–88.

[B44] Elegbede C. F. Pierrat J. C. Aguayo J. Husson C. Halkett F. Marçais B. A. (2010). Statistical model to detect asymptomatic infectious individuals with an application in the *Phytophthora alni*-induced alder decline. Phytopathology 100, 1262–1269. doi: 10.1094/PHYTO-05-10-0140 20932169

[B45] Eschen R. Rigaux L. Sukovata L. Vettraino A. M. Marzano M. Grégoire J.-C. (2015). Phytosanitary inspection of woody plants for planting at European Union entry points: a practical enquiry. Biol. Invasions 17, 2403–2413. doi: 10.1007/s10530-015-0883-6

[B46] Fan S. Georgi L. L. Hebard F. V. Zhebentyayeva T. Yu J. Sisco P. H.. (2024). Mapping QTLs for blight resistance and morpho-phenological traits in inter-species hybrid families of chestnut (*Castanea* spp.). Front. Plant Sci. 8. doi: 10.3389/fpls.2024.1365951 PMC11033410 38650705

[B47] Feio M. J. Alves T. Boavida M. Medeiros A. Graça M. A. S. (2010). Functional indicators of stream health: ariver-basin approach. Freshw. Biol. 55, 1050–1065. doi: 10.1111/j.1365-2427.2009.02332.x

[B48] Ferentinos K. (2018). Deep learning models for plant disease detection and diagnosis. Comp. Electron. Agr. 145, 311–318. doi: 10.1016/j.compag.2018.01.009

[B49] Fernandes P. Colavolpe M. B. Serrazina S. Costa R. L. (2022). European and American chestnuts: an overview of the main threats and control efforts. Front. Plant Sci. 13. doi: 10.3389/fpls.2022.951844 PMC9449730 36092400

[B50] Fernandes P. Pimentel D. Ramiro R. S. do Céu Silva M. Fevereiro P. Costa R. L. (2024). Dual transcriptomic analysis reveals early induced *Castanea* genes and *Phytophthora cinnamomi* effectors. Front. Plant Sci. 15. doi: 10.3389/fpls.2024.1439380 PMC11345161 39188543

[B51] Ferreira V. Encalada A. C. Graça M. A. S. (2012). Effects of litter diversity on decomposition and biological colonization of submerged litter in temperate and tropical streams. Freshw. Sci. 31, 945–962. doi: 10.1899/11-062.1

[B52] Ferreira V. Pazianoto L. H. Solla A. (2022). Invasive forest pathogens affect the characteristics, microbial colonisation, and decomposition of leaf litter in streams. Freshw. Biol. 67, 416–429. doi: 10.1111/fwb.13851

[B53] Fisher M. C. Gurr S. J. Cuomo C. A. Blehert D. S. Jin H. Stukenbrock E. H.. (2020). Threats posed by the fungal kingdom to humans, wildlife, and agriculture. mBio 11. doi: 10.1128/mBio.00449-20 PMC7403777 32371596

[B54] Fuller E. Germaine K. J. Rathore D. S. (2023). The good, the bad, and the useable microbes within the common alder (*Alnus glutinosa*) microbiome-potential bio-agents to combat alder dieback. Microorganisms 11, 2187. doi: 10.3390/microorganisms11092187 37764031 PMC10535473

[B55] Gibbs J. N. Lipscombe M. A. Peace A. J. (1999). The impact of *Phytophthora* disease on riparian populations of common alder (*Alnus glutinosa*) in southern Britain. Eur. J. For. Pathol. 29, 39–50. doi: 10.1046/j.1439-0329.1999.00129.x

[B56] Gibbs J. N. Van Dijk C. Webber J. F. (2003). Phytophthora disease of alder in Europe. Forestry Commission Bulletin 126 (Edinburgh, UK: Forestry Commission).

[B57] Gomes Marques I. Solla A. David T. S. Rodríguez-González P. M. Garbelotto M. (2022). Response of two riparian woody plants to *Phytophthora* species and drought. For. Ecol. Manage. 518, 120281. doi: 10.1016/j.foreco.2022.120281

[B58] Gomes Marques I. Vieites-Blanco C. Barrento M. J. Semedo J. N. Rodrigues A. P. Scotti-Campos P.. (2024a). Phenotypic variation and genetic diversity in European *Alnus* species. Forestry, cpae039. doi: 10.1093/forestry/cpae039

[B59] Gomes Marques I. Vieites-Blanco C. Rodríguez-González P. M. Segurado P. Marques M. Barrento M. J.. (2024b). The ADnet Bayesian belief network for alder decline: Integrating empirical data and expert knowledge. Sci. Total Environ. 947, 173619. doi: 10.1016/j.scitotenv.2024.173619 38825208

[B60] Gomes Marques I. (2024). Resilience of alder in response to global change stressors. PhD thesis. School of Agriculture, University of Lisbon.

[B61] Graça M. A. S. Cressa C. (2010). Leaf quality of some tropical and temperate tree species as food resource for stream shredders. Int. Rev. Hydrobiol. 95, 27–41. doi: 10.1002/iroh.200911173

[B62] Greenwood M. S. Weir R. J. (1995). Genetic variation in rooting ability of loblolly pine cuttings: effect of auxin and family on rooting by hypocotyl cuttings. Tree Physiol. 15, 41–45. doi: 10.1093/treephys/15.1.41 14966010

[B63] Gryta H. Van de Paer C. Manzi S. Holota H. Roy M. Besnard G. (2017). Genome skimming and plastid microsatellite profiling of alder trees (*Alnus* spp., Betulaceae): phylogenetic and phylogeographical prospects. Tree Genet. Genomes 13, 1–14. doi: 10.1007/s11295-017-1204-2

[B64] Guerra-Hernández J. Díaz Varela R. A. Álvarez González J. G. Rodríguez González P. M. (2021). Assessing a novel modelling approach with high resolution UAV imagery for monitoring health status in priority riparian forests. For. Ecosyst. 61, 8. doi: 10.1186/s40663-021-00342-8

[B65] Guignard L. Sauvageau C. (1894). Sur un nouveau microbe chromogene, le *Bacillus chlororaphis.* C. R. Soc Biol. Paris Ser. 10 1, 841–843.

[B66] Hamilton J. A. Royauté R. Wright J. W. Hodgskiss P. Ledig F. T. (2017). Genetic conservation and management of the California endemic, Torrey pine (*Pinus torreyana* Parry): Implications of genetic rescue in a genetically depauperate species. Ecol. Evol. 7, 7370–7381. doi: 10.1002/ece3.3306 28944023 PMC5606898

[B67] Handa I. T. Aerts R. Berendse F. Berg M. P. Bruder A. Butenschoen O.. (2014). Consequences of biodiversity loss for litter decomposition across biomes. Nature 509, 218–221. doi: 10.1038/nature13247 24805346

[B68] Hansen E. M. Reeser P. Sutton W. Sniezko R. A. (2012). “Methods for screening Port-Orford-Cedar for resistance to *Phytophthora lateralis* ,” in Proceedings of the fourth international workshop on the genetics of host-parasite interactions in forestry: disease and insect resistance in forest trees. Trees. Gen. Tech. Rep. PSW-GTR-240 (Pacific Southwest Research Station, Forest Service, U.S. Department of Agriculture, Albany, CA), 181–188.

[B69] Haque M. M. Martínez-Álvarez P. Lomba J. M. Martín-García J. Diez J. J. (2014). First report of *Phytophthora plurivora* causing collar rot on common alder in Spain. Plant Dis. 98, 425–425. doi: 10.1094/PDIS-07-13-0784-PDN 30708423

[B70] Haque M. M. U. Martín-García J. Diez J. J. (2015). Variation in pathogenicity among the three subspecies of *Phytophthora alni* on detached leaves, twigs and branches of *Alnus glutinosa.* For. Pathol. 45, 484–491. doi: 10.1111/efp.12198

[B71] Havrdová A. Douda J. Krak K. Vít P. Hadincová V. Zákravský P.. (2015). Higher genetic diversity in recolonized areas than in refugia of *Alnus glutinosa* triggered by continent-wide lineage admixture. Mol. Ecol. 24, 4759–4777. doi: 10.1111/efp.12239 26290117

[B72] Herms D. A. McCullough D. G. (2014). Emerald ash borer invasion of North America: history, biology, ecology, impacts, and management. Annu. Rev. Entomol. 59, 13–30. doi: 10.1146/annurev-ento-011613-162051 24112110

[B73] Horta Jung M. Maia C. Mora-Sala B. Abad-Campos P. Schena L. Mosca S.. (2024). High diversity of *Phytophthora* species in natural ecosystems and nurseries of Portugal: Detrimental side effect of plant introductions from the age of discovery to modern globalization. Plant Pathol. 00, 1–33. doi: 10.1111/ppa.14022

[B74] Huff M. Seaman J. Wu D. Zhebentyayeva T. Kelly L. J. Faridi N.. (2022). A high-quality reference genome for *Fraxinus pennsylvanica* for ash species restoration and research. Mol. Ecol. Resour. 22, 1284–1302. doi: 10.1111/1755-0998.13545 34748273 PMC9299157

[B75] Husson C. Aguayo J. Revellin C. Frey P. Ioos R. Marçais B. (2015). Evidence for homoploid speciation in *Phytophthora alni* supports taxonomic reclassification in this species complex. Fungal Genet. Biol. 77, 12–21. doi: 10.1016/j.fgb.2015.02.013 25732380

[B76] Jabiol J. Lecerf A. Lamothe S. Gessner M. O. Chauvet E. (2019). Litter quality modulates effects of dissolved nitrogen on leaf decomposition by stream microbial communities. Microb. Ecol. 77, 959–966. doi: 10.1007/s00248-019-01353-3 30899980

[B77] Jacobs D. C. Revord R. S. Capik J. M. Mehlenbacher S. A. Thomas J. M. (2024). Variable response of eastern filbert blight resistance sources in New Jersey. Front. Plant Sci. 15. doi: 10.3389/fpls.2024.1419265 PMC11303342 39114467

[B78] Jung T. Blaschke M. (2004). *Phytophthora* root and collar rot of alders in Bavaria: distribution, modes of spread and possible management strategies. Plant Pathol. 53, 197–208. doi: 10.1111/j.0032-0862.2004.00957.x

[B79] Jung T. Burgess T. I. (2009). Re-evaluation of *Phytophthora citricola* isolates from multiple woody hosts in Europe and North America reveals a new species, Phytophthora plurivora sp. nov. Persoonia - Mol. Phylogeny Evol. Fungi 22, 95–110. doi: 10.3767/003158509X442612 PMC2789536 20198142

[B80] Jung T. Cech T. Vannini A. (2013). “The impact of invasive *Phytophthora* species on European forests,” in *Phytophthora*: a global perspective. Ed. Lamour K. (CABI Digital Library), 146–158. doi: 10.1079/9781780640938.0146

[B81] Jung T. Orlikowski L. Henricot B. Abad-Campos P. Aday A. G. Aguín Casal O.. (2016). Widespread *Phytophthora* infestations in European nurseries put forest, semi-natural and horticultural ecosystems at high risk of *Phytophthora* diseases. For. Pathol. 46, 134–163. doi: 10.1111/efp.12239

[B82] Jung T. Pérez-Sierra A. Durán A. Jung M. H. Balci Y. Scanu B. (2018). Canker and decline diseases caused by soil-and airborne *Phytophthora* species in forests and woodlands. Persoonia: Mol. Phylogeny Evol. Fungi 40, 182–220. doi: 10.3767/persoonia.2018.40.08 PMC6146643 30505001

[B83] Kanoun-Boulé M. Vasconcelos T. Gaspar J. Vieira S. Dias-Ferreira C. Husson C. (2016). *Phytophthora × alni* and *Phytophthora lacustris* associated with common alder decline in Central Portugal. For. Pathol. 46, 174–176. doi: 10.1111/efp.12273

[B84] Kelly L. J. Plumb W. J. Carey D. W. Mason M. E. Cooper E. D. Crowther W.. (2020). Convergent molecular evolution among ash species resistant to the emerald ash borer. Nat. Ecol. Evol. 4, 1116–1128. doi: 10.1038/s41559-020-1209-3 32451426 PMC7610378

[B85] Keriö S. Daniels H. A. Gómez-Gallego M. Tabima J. F. Lenz R. R. Søndreli K. L.. (2019). From genomes to forest management – tackling invasive *Phytophthora* species in the era of genomics. Can. J. Plant Pathol. 42, 1–29. doi: 10.1080/07060661.2019.1626910

[B86] Lane T. Best T. Zembower N. Davitt J. Henry N. Xu Y.. (2016). The green ash transcriptome and identification of genes responding to abiotic and biotic stresses. BMC Genomics 17, 702. doi: 10.1186/s12864-016-3052-0 27589953 PMC5009568

[B87] Lee B. D. Dutta S. Ryu H. Yoo S. J. Suh D. S. Park K. (2015). Induction of systemic resistance in *Panax ginseng* against *Phytophthora cactorum* by native *Bacillus amyloliquefaciens* HK34. J. Ginseng Res. 39, 213–220. doi: 10.1016/j.jgr.2014.12.002 26199552 PMC4506372

[B88] Lee J. H. Pijut P. M. (2018). Optimization of *Agrobacterium*-mediated genetic transformation of *Fraxinus nigra* and development of black ash for possible emerald ash borer resistance. Plant Cell Tiss. Organ Cult. 134, 217–229. doi: 10.1007/s11240-018-1414-9

[B89] Lenz P. R. N. Nadeau S. Mottet M.-J. Perron M. Isabel N. Beaulieu J.. (2020). Multi-trait genomic selection for weevil resistance, growth, and wood quality in Norway spruce. Evol. Appl. 13, 76–94. doi: 10.1111/eva.12823 31892945 PMC6935592

[B90] Lepais O. Muller S. Ben Saad-Limam S. Benslama M. Rhazi L. Belouahem-Abed D.. (2013). High genetic diversity and distinctiveness of rear-edge climate relicts maintained by ancient tetraploidisation for *Alnus glutinosa* . PLoS One 8, e75029. doi: 10.1371/JOURNAL.PONE.0075029 24098677 PMC3787099

[B91] Li W. Zheng X. Cheng R. Zhong C. Zhao J. Liu T. H.. (2023). Soybean ZINC FINGER PROTEIN03 targets two SUPEROXIDE DISMUTASE1s and confers resistance to *Phytophthora sojae* . Plant Physiol. 192, 633–647. doi: 10.1093/plphys/kiad083 36782397 PMC10152685

[B92] Lima R. P. M. Máximo H. J. Merfa M. V. Dalio R. J. D. Cristofani-Yaly M. MaChado M. A. (2018). Genetic tools and strategies for citrus breeding aiming at resistant rootstocks to gummosis disease. Trop. Plant Pathol. 43, 279–288. doi: 10.1007/s40858-018-0229-x

[B93] Lin Y.-C. Mansfeld B. N. Tang X. Colle M. Chen F. Weng Y.. (2023). Identification of QTL associated with resistance to *Phytophthora* fruit rot in cucumber (*Cucumis sativus* L.). Front. Plant Sci. 14. doi: 10.3389/fpls.2023.1281755 PMC10693349 38046614

[B94] Macháčová M. Tomášková I. Corcobado T. Nagy Z. Milanović S. Janoušek J.. (2024). Response of *Alnus glutinosa* to *Phytophthora* bark infections at ambient and elevated CO_2_ levels. Front. For. Glob. Change 7. doi: 10.3389/ffgc.2024.1379791

[B95] Mamidala P. Wijeratne A. J. Wijeratne S. Poland T. Qazi S. S. Doucet D.. (2013). Identification of odor-processing genes in the emerald ash borer, *agrilus planipennis* . PLoS One 8, e56555. doi: 10.1371/journal.pone.0056555 23424668 PMC3570424

[B96] Mandák B. Vít P. Krak K. Trávníček P. Havrdová A. Hadincová V.. (2016). Flow cytometry, microsatellites and niche models reveal the origins and geographical structure of *Alnus glutinosa* populations in Europe. Ann. Bot. 117, 107–120. doi: 10.1093/AOB/MCV158 26467247 PMC4701152

[B97] Martín J. A. López R. (2023). Biological deterioration and natural durability of wood in Europe. Forests 14, 283. doi: 10.3390/f14020283

[B98] Martín M. A. Moreno R. Die J. V. Cabrera A. Castro P. Pérez M. D.. (2024). Distribution, diversity and genetic structure of alders (*Alnus lusitanica* and A. glutinosa) in Spain. For. Ecol. Manage. 562, 121922. doi: 10.1016/j.foreco.2024.121922

[B99] Martínez M. T. Cuenca B. Mosteiro F. Piñeiro P. Pérez F. Solla A.. (2023). Screening of cork oak for resistance to *Phytophthora cinnamomi* and micropropagation of tolerant seedlings. Horticulturae 9, 692. doi: 10.3390/horticulturae9060692

[B100] Martín-García J. Solla A. Corcobado T. Siasou E. Woodward S. (2015). Influence of temperature on germination of *Quercus ilex* in *Phytophthora cinnamomi*, P. gonapodyides, *P. quercina* and *P. psychrophila* infested soils. For. Pathol. 45, 215–223. doi: 10.1111/efp.12159

[B101] Menéndez-Gutiérrez M. Alonso M. Díaz R. (2021). Assessing genetic variation in resistance to pinewood nematode (*Bursaphelenchus xylophilus*) in *Pinus radiata* D. Don half-sib families. Forests 12, 1474. doi: 10.3390/f12111474

[B102] Menéndez-Gutiérrez M. Alonso M. Toval G. Díaz R. (2018). Testing of selected *Pinus pinaster* half-sib families for tolerance to pinewood nematode (*Bursaphelenchus xylophilus*). Forestry: Int. J. For. Res. 91, 38–48. doi: 10.1093/forestry/cpx030

[B103] Mercado-Blanco J. Abrantes I. Barra Caracciolo A. Bevivino A. Ciancio A. Grenni P.. (2018). Belowground microbiota and the health of tree crops. Front. Microbiol. 9. doi: 10.3389/fmicb.2018.01006 PMC5996133 29922245

[B104] Merkle S. A. Koch J. L. Tull A. R. Dassow J. E. Carey D. W. Barnes B. F.. (2023). Application of somatic embryogenesis for development of emerald ash borer-resistant white ash and green ash varietals. New For. 54, 697–720. doi: 10.1007/s11056-022-09903-3 PMC8933133 35344318

[B105] Mingeot D. Baleux R. Watillon B. (2010). Characterization of microsatellite markers for black alder (*Alnus glutinosa* [L.] Gaertn). Conserv. Genet. Resour. 2, 269–271. doi: 10.1007/s12686-010-9188-3

[B106] Miranda-Fontaíña M. E. Fernández-López J. Vettraino A. M. Vannini A. (2007). Resistance of *Castanea* clones to *Phytophthora cinnamomi*: testing and genetic control. Silvae Genetica 56, 11–21. doi: 10.1515/sg-2007-0002

[B107] Molinero J. Pozo J. (2006). Organic matter, nitrogen and phosphorus fluxes associated with leaf litter in two small streams with different riparian vegetation: a budget approach. Arch. Hydrobiol. 166, 363–386. doi: 10.1127/0003-9136/2006/0166-0363

[B108] Mora-Sala B. León M. Pérez-Sierra A. Abad-Campos P. (2022). New reports of *Phytophthora* species in plant nurseries in Spain. Pathogens 11, 826. doi: 10.3390/pathogens11080826 35894049 PMC9394253

[B109] Mpika J. Kébé I. B. Issali A. E. N’guessan F. K. Druzhinina S. Komon-Zélazowska M.. (2009). Antagonist potential of *Trichoderma indigenous* isolates for biological control of *Phytophthora palmivora* the causative agent of black pod disease on cocoa (*Theobroma cacao* L.) in Côte d’Ivoire. Afr. J. Biotechnol. 8, 5280–5293. doi: 10.4314/ajb.v8i20.65962

[B110] Narayan H. Srivasatava P. Chandra Bhatt S. Joshi D. Soni R. (2022). “Plant pathogenesis and disease control,” in Plant Protection: From Chemicals to Biologicals. Eds. Soni R. Suyal D. C. Goel R. (De Gruyter, Berlín, Boston, MA), 95–114. doi: 10.1515/9783110771558-005

[B111] Nave C. Schwan J. Werres S. Riebesehl J. (2021). *Alnus glutinosa* threatened by alder *Phytophthora*: a histological study of roots. Pathogens 10, 977. doi: 10.3390/pathogens10080977 34451441 PMC8401901

[B112] Neale D. B. Kremer A. (2011). Forest tree genomics: growing resources and applications. Nat. Rev. Genet. 12, 111–122. doi: 10.1038/nrg2931 21245829

[B113] Nie X. Zhao S. Hao Y. Gu S. Zhang Y. Qi B.. (2023). Transcriptome analysis reveals key genes involved in the resistance to *Cryphonectria parasitica* during early disease development in Chinese chestnut. BMC Plant Biol. 23, 79. doi: 10.1186/s12870-023-04072-7 36740701 PMC9901152

[B114] Novotná K. Štochlová P. (2012). Selection of the best method for vegetative propagation of mature *Alnus glutinosa* (L.) Gaertn. trees resistant to *Phytophthora alni* . Acta Univ. Agric. silvic. Mendel. Brun. 60, 105–110. doi: 10.11118/actaun201260010105

[B115] Oßwald W. Fleischmann F. Rigling D. Coelho A. C. Cravador A. Diez J.. (2014). Strategies of attack and defence in woody plant–*Phytophthora* interactions. For. Path. 44, 169–190. doi: 10.1111/efp.12096

[B116] Pérez F. Cuenca B. Ruíz-Gómez F. J. Rey M. D. Galea M. R. Arrillaga I.. (2020). Programa de mejora y conservación de los recursos genéticos de la encina y el alcornoque frente al síndrome de la seca. Foresta 78, 56–61.

[B117] Périnet F. Tremblay F. M. (1987). Commercial micropropagation of five *Alnus* species. New For. 3, 225–230. doi: 10.1007/BF00118760

[B118] Pintos-Varela C. Martínez C. R. Aguín Casal O. Vázquez J. P. M. Yebra A. A. (2012). First report of *Phytophthora alni* subsp. *uniformis* on black alder in Spain. Plant Dis. 96, 589. doi: 10.1094/PDIS-10-11-0891-PDN 30727424

[B119] Pintos-Varela C. Rial C. Aguín O. Ferreiroa V. Mansilla J. P. (2016). First report of *Phytophthora hydropathica* in river water associated with riparian alder in Spain. New Dis. Rep. 33, 25. doi: 10.5197/j.2044-0588.2016.033.025

[B120] Pintos-Varela C. Rial-Martínez C. Aguín-Casal O. Mansilla-Vázquez J. P. (2017). First report of *Phytophthora × multiformis* on *Alnus glutinosa* in Spain. Plant Dis. 101, 261. doi: 10.1094/PDIS-08-16-1092-PDN 30754290

[B121] Pintos-Varela C. Rial Martínez C. Mansilla Vázquez J. P. Aguín Casal O. (2010). First report of *Phytophthora* rot on alders caused by *Phytophthora alni* subsp. *alni* in Spain. Plant Dis. 94, 273. doi: 10.1094/PDIS-94-2-0273A 30754290

[B122] Poland T. M. McCullough D. G. (2006). Emerald ash borer: invasion of the urban forest and the threat to North America’s ash resource. J. For. 104, 118–124. doi: 10.1093/jof/104.3.118

[B123] Poovaiah C. Phillips L. Geddes B. Reeves C. Sorieul M. Thorlby G. (2021). Genome editing with CRISPR/Cas9 in *Pinus radiata* (D. Don). BMC Plant Biol. 21, 363. doi: 10.1186/s12870-021-03143-x 34376154 PMC8353756

[B124] Portela-Pereira E. Monteiro P. Rodríguez-González P. M. (2022). Control of invasive plant species in wetland forests (91E0*). Biol. Life Sci. Forum 13, 84. doi: 10.3390/blsf2022013084

[B125] Pozo J. González E. Díez J. R. Molinero J. Elósegui A. (1997). Inputs of particulate organic matter to streams with different riparian vegetation. J. N. Am. Benthol. Soc 16, 602–611. doi: 10.2307/1468147

[B126] Quintana-Rodríguez E. Morales-Vargas A. T. Molina-Torres J. Ádame-Alvarez R. M. Acosta-Gallegos J. A. Heil M. (2015). Plant volatiles cause direct, induced and associational resistance in common bean to the fungal pathogen *Colletotrichum lindemuthianum* . J. Ecol. 103, 250–260. doi: 10.1111/1365-2745.12340

[B127] Radwan M. A. Max T. A. Johnson D. W. (1989). Softwood cuttings for propagation of red alder. New For. 3, 21–30. doi: 10.1007/BF00128898

[B128] Rairdin A. Fotouhi F. Zhang J. Mueller D. S. Ganapathysubramanian B. Singh A. K.. (2022). Deep learning-based phenotyping for genome wide association studies of sudden death syndrome in soybean. Front. Plant Sci. 13. doi: 10.3389/fpls.2022.966244 PMC9634489 36340398

[B129] Redondo M. A. Boberg J. Olsson C. H. B. Oliva J. (2015b). Winter conditions correlate with *Phytophthora alni* subspecies distribution in southern Sweden. Phytopathology 105, 1191–1197. doi: 10.1094/PHYTO-01-15-0020-R 25822186

[B130] Redondo M. A. Pérez-Sierra A. Abad-Campos P. Torres L. Solla A. Reig-Armiñana J.. (2015a). Histology of *Quercus ilex* roots during infection by *Phytophthora cinnamomi* . Trees 29, 1943–1957. doi: 10.1007/s00468-015-1275-3

[B131] Redondo M. A. Stenlid J. Oliva J. (2020). Genetic variation explains changes in susceptibility in a naïve host against an invasive forest pathogen: the case of alder and the *Phytophthora alni* complex. Phytopathology 110, 517–525. doi: 10.1094/PHYTO-07-19-0272-R 31552784

[B132] Rhoades C. Oskarsson H. Binkley D. Stottlemyer B. (2001). Alder (*Alnus crispa*) effects on soils in ecosystems of the Agashashok River valley, northwest Alaska. Ecoscience 8, 89–95. doi: 10.1080/11956860.2001.11682634

[B133] Rial-Martínez C. Souto-Herrero M. Piñón-Esteban P. García-González I. Aguín-Casal O. Salinero-Corral C.. (2023). First report of root rot caused by *Phytophthora lacustris* on alder (*Alnus lusitanica)* in Spain. Plant Dis. 107, 3322. doi: 10.1094/PDIS-04-23-0793-PDN

[B134] Ro N. Haile M. Hur O. Geum B. Rhee J. Hwang A.. (2022). Genome-wide association study of resistance to *Phytophthora capsici* in the pepper (*Capsicum* spp.) collection. Front. Plant Sci. 13. doi: 10.3389/fpls.2022.902464 PMC9164128 35668797

[B135] Rodrigues da Silva A. da Costa Silva D. Dos Santos Pinto K. N. Santos Filho H. P. Coelho Filho M. A. Dos Santos Soares Filho W.. (2021). Epigenetic responses to *Phytophthora citrophthora* gummosis in citrus. Plant Sci. 313, 111082. doi: 10.1016/j.plantsci.2021.111082 34763867

[B136] Rodriguez R. Diíaz-Sala C. Cuozzo L. Ancora G. (1991). Pear in *vitro* propagation using a double-phase culture system. HortScience 26, 62–64. doi: 10.21273/HORTSCI.26.1.62

[B137] Rodríguez-González P. M. García C. Albuquerque A. Monteiro-Henriques T. Faria C. Guimarães J. B.. (2019). A spatial stream-network approach assists in managing the remnant genetic diversity of riparian forests. Sci. Rep. 9, 6741. doi: 10.1038/s41598-019-43132-7 31043695 PMC6494995

[B138] Rodríguez-González P. M. Stella J. C. Campelo F. Ferreira M. T. Albuquerque A. (2010). Subsidy or stress? Tree structure and growth in wetland forests along a hydrological gradient in Southern Europe. For. Ecol. Manage. 259, 2015–2025. doi: 10.1016/j.foreco.2010.02.012

[B139] Rubio-Ríos J. Salinas-Bonillo M. J. Pérez J. Fenoy E. Boyero L. Casas J. J. (2023). Alder stands promote N-cycling but not leaf litter mass loss in Mediterranean streams flowing through pine plantations. For. Ecol. Manage. 542, 121072. doi: 10.1016/j.foreco.2023.121072

[B140] San José M. C. Blázquez N. Cernadas M. J. Janeiro L. V. Cuenca B. Sánchez C.. (2020). Temporary immersion systems to improve alder micropropagation. Plant Cell Tiss. Organ Cult. 143, 265–275. doi: 10.1007/s11240-020-01937-9

[B141] San José M. C. Corredoira E. Janeiro L. V. (2011). Efecto de los carbohidratos sobre la micropropagacion de *Alnus glutinosa* (L.) Gaertn. Span. J. Rural Dev. 2, 9–18. doi: 10.5261/2011.GEN4.02

[B142] San José M. D. C. Corredoira E. Oliveira H. Santos C. (2015b). Cryopreservation of somatic embryos of *Alnus glutinosa* (L.) Gaertn. and confirmation of ploidy stability by flow cytometry. Plant Cell Tiss. Org. Cult. 123, 489–499. doi: 10.1007/s11240-015-0853-9

[B143] San José M. C. Janeiro L. V. Corredoira E. (2013). Micropropagation of threatened black alder. Silva Fennica 47, 892. doi: 10.14214/sf.892

[B144] San José M. C. Janeiro L. V. Corredoira E. (2015a). Simple strategy for the in *vitro* conservation of *Alnus glutinosa* (L.) Gaertn. germplasm. Trees-Struct. Funct. 29, 539–549. doi: 10.1007/s00468-014-1133-8

[B145] San José M. C. Romero L. Janeiro L. V. (2012). Effect of indole-3-butyric acid on root formation in *Alnus glutinosa* microcuttings. Silva Fenn. 46, 643–654. doi: 10.14214/sf.916

[B146] Sanna M. González Toral C. Nava H. S. Cuesta C. Loidi J. Herrera M.. (2023). Contribution to the knowledge of the distribution of *Alnus* species in southern Europe based on cpDNA. Nat. Cantab. 11, 41–52.

[B147] Santos C. MaChado H. Correia I. Gomes F. Gomes-Laranjo J. Costa R. (2015). Phenotyping *Castanea* hybrids for *Phytophthora cinnamomi* resistance. Plant Pathol. 64, 901–910. doi: 10.1111/ppa.12313

[B148] Santos C. Nelson C. D. Zhebentyayeva T. MaChado H. Gomes-Laranjo J. Costa R. L. (2017). First interspecific genetic linkage map for *Castanea sativa* x *Castanea crenata* revealed QTLs for resistance to *Phytophthora cinnamomi* . PLoS One 12, e0184381. doi: 10.1371/journal.pone.0184381 28880954 PMC5589223

[B149] Schmitt U. Singh A. P. Kim Y. S. (2021). “Wood as an ecological niche for microorganisms: wood formation, structure, and cell wall composition,” in Forest Microbiology: Tree Microbiome - Phyllosphere, Endosphere and Rhizosphere. Eds. Asiegbu F. O. Kovalchuk A. (Elsevier, San Diego, CA, USA), 17–34. doi: 10.1016/B978-0-12-822542-4.00010-3

[B150] Shaftel R. S. King R. S. Back J. A. (2012). Alder cover drives nitrogen availability in Kenai lowland headwater streams, Alaska. Biogeochemistry 107, 135–148. doi: 10.1007/s10533-010-9541-3

[B151] Shainsky L. J. Rose C. L. (1995). Effects of competition on the foliar chemistry of young Douglas-fir in monoculture and mixed stands with young red alder. Can. J. For. Res. 25, 1969–1977. doi: 10.1139/x95-212

[B152] Sharma U. Sankhyan H. P. Kumari A. Thakur S. Thakur L. Mehta D.. (2024). Genomic selection: a revolutionary approach for forest tree improvement in the wake of climate change. Euphytica 220, 9. doi: 10.1007/s10681-023-03263-5

[B153] Shen S. Fan Z. Zhang X. Kong X. Liu F. Zhang Z.. (2021). The characteristics of chemosensory and opsin genes in newly emerged and sexually mature *Agrilus planipennis*, an important quarantine forest beetle. Front. Genet. 11. doi: 10.3389/fgene.2020.604757 PMC7844324 33519910

[B154] Siddique M. I. Lee H. Y. Ro N. Y. Han K. Venkatesh J. Solomon A. M.. (2019). Identifying candidate genes for *Phytophthora capsici* resistance in pepper (*Capsicum annuum*) via genotyping-by-sequencing-based QTL mapping and genome-wide association study. Sci. Rep. 9, 9962. doi: 10.1038/s41598-019-46342-1 31292472 PMC6620314

[B155] Siviero A. Cristofani M. Furtado E. L. Garcia A. A. Coelho A. S. MaChado M. A. (2006). Identification of QTLs associated with citrus resistance to *Phytophthora gummosis* . J. Appl. Genet. 47, 23–28. doi: 10.1007/BF03194595 16424605

[B156] Sjöman H. Watkins H. Kelly L. J. Hirons A. Kainulainen K. Martin K. W. E.. (2024). Resilient trees for urban environments: the importance of intraspecific variation. Plants People Planet 6, 1180–1189. doi: 10.1002/ppp3.10518

[B157] Smeriglio A. D’Angelo V. Cacciola A. Ingegneri M. Raimondo F. M. Trombetta D.. (2022). New insights on phytochemical features and biological properties of *Alnus glutinosa* stem bark. Plants 11, 2499. doi: 10.3390/plants11192499 36235365 PMC9570633

[B158] Šmíd J. Douda J. Krak K. Mandák B. (2020). Analyses of hybrid viability across a hybrid zone between two Alnus species using microsatellites and cpDNA markers. Genes 11, 770. doi: 10.3390/GENES11070770 32659930 PMC7397206

[B159] Sniezko R. A. (2006). Resistance breeding against nonnative pathogens in forest trees: current successes in North America. Can. J. Plant Pathol. 28, S270–S279. doi: 10.1080/07060660609507384

[B160] Sniezko R. A. Koch J. (2017). Breeding trees resistant to insects and diseases: putting theory into application. Biol. Invasions 19, 3377–3400. doi: 10.1007/s10530-017-1482-5

[B161] Solla A. Pérez-Sierra A. Corcobado T. Haque M. M. Diez J. J. Jung T. (2010). *Phytophthora alni* on *Alnus glutinosa* reported for the first time in Spain. Plant Pathol. 59, 798–798. doi: 10.1111/j.1365-3059.2009.02254.x

[B162] Sollars E. Harper A. Kelly L. Sambles C. M. Ramirez-Gonzalez R. H. Swarbreck D.. (2017). Genome sequence and genetic diversity of European ash trees. Nature 541, 212–216. doi: 10.1038/nature20786 28024298

[B163] Spalding E. P. Miller N. D. (2013). Image analysis is driving a renaissance in growth measurement. Curr. Opin. Plant Biol. 16, 100–104. doi: 10.1016/j.pbi.2013.01.001 23352714

[B164] Stanley R. K. Carey D. W. Mason M. E. Doran A. Wolf J. Otoo K. O.. (2023). Emerald ash borer (*Agrilus planipennis*) infestation bioassays and metabolic profiles of green ash (*Fraxinus pennsylvanica*) provide evidence for an induced host defensive response to larval infestation. Front. For. Glob. Change 6. doi: 10.3389/ffgc.2023.1166421

[B165] Steddom K. Becker O. Menge J. A. (2002). Repetitive applications of the biocontrol agent *Pseudomonas putida* 06909-rif/nal and effects on populations of *Phytophthora parasitica* in citrus orchards. Phytopathology 92, 850–856. doi: 10.1094/PHYTO.2002.92.8.850 18942963

[B166] Štochlová P. Novotná K. Černý K. (2012). Factors affecting the development of Phytophthora alni ssp. *aln*i infections in *Alnus glutinosa* L. J. For. Sci. 58, 123–130. doi: 10.17221/26/2011-JFS

[B167] Tapias R. Fernández M. Moreira A. C. Sánchez E. Cravador A. (2006). “Posibilidades de la variabilidad genética de encinas y alcornoques en la conservación y recuperación de bosques amenazados por la “seca”,” in Boletín informativo CIDEU. Ed. Tapias R. (Huelva, Spain: CIDEU-Universidad de Huelva), 45–51.

[B168] Tapias R. Moreira A. C. Fernández M. Saenz A. Domingos A. C. Melo E.. (2008). “Variability in the tolerance/resistance of *Quercus suber* L. seedlings to *Phytophthora cinnamomi* Rands: evaluation of survival,” in Suberwood: new challenges for the integration of cork oak forests and products. Eds. Vázquez-Piqué J. Pereira H. González-Pérez A. (Huelva, Spain: Universidad de Huelva Publicaciones), 237–246.

[B169] Teklehaimanot Z. Mmolotsi R. M. (2007). Contribution of red alder to soil nitrogen input in a silvopastoral system. Biol. Fert. Soils 43, 843–848. doi: 10.1007/s00374-006-0163-9

[B170] Teshome D. T. Zharare G. E. Naidoo S. (2020). The threat of the combined effect of biotic and abiotic stress factors in forestry under a changing climate. Front. Plant Sci. 11. doi: 10.3389/fpls.2020.601009 PMC7733969 33329666

[B171] Tian L. Lin X. Tian J. Ji L. Chen Y. Tran L.-S. P.. (2020). Research advances of beneficial microbiota associated with crop plants. Int. J. Mol. Sci. 21, 1792. doi: 10.3390/ijms21051792 32150945 PMC7084388

[B172] Tremblay É.D. Duceppe M.-O. Bérubé J. A. Kimoto T. Lemieux C. Bilodeau G. J. (2018). Screening for exotic forest pathogens to increase survey capacity using metagenomics. Phytopathology 108, 1509–1521. doi: 10.1094/PHYTO-02-18-0028-R 29923801

[B173] Tremblay F. M. Lalonde M. (1984). Requirements for in *vitro* propagation of seven nitrogen-fixing *Alnus* species. Plant Cell Tiss. Organ Cult. 3, 189–199. doi: 10.1007/BF00040337

[B174] Trzewik A. Maciorowski R. Orlikowska T. (2021). Pathogenicity of *Phytophthora× alni* isolates obtained from symptomatic trees, soil and water against alder. Forests 13, 20. doi: 10.3390/f13010020

[B175] Tunyasuvunakool K. Adler J. Wu Z. Green T. Zielinski M. Žídek A.. (2021). Highly accurate protein structure prediction for the human proteome. Nature 596, 590–596. doi: 10.1038/s41586-021-03828-1 34293799 PMC8387240

[B176] van den Berg N. Christie J. B. Aveling T. A. S. Engelbrecht J. (2018). Callose and β-1,3-glucanase inhibit *Phytophthora cinnamomi* in a resistant avocado rootstock. Plant Pathol. 67, 1150–1160. doi: 10.1111/ppa.12819

[B177] Vieites-Blanco C. Colangelo M. Camarero J. J. Caballol M. García Breijo F. J. Štraus D.. (2023). Pathogenicity of *Phytophthora* and *Halophytophthora* species on black alder and the host histological response. Mycol. Prog. 22, 71. doi: 10.1007/s11557-023-01923-3

[B178] Vít P. Douda J. Krak K. Havrdová A. Mandák B. (2017). Two new polyploid species closely related to *Alnus glutinosa* in Europe and North Africa – an analysis based on morphometry, karyology, flow cytometry and microsatellites. Taxon 66, 567–583. doi: 10.12705/663.4

[B179] Vörösmarty C. McIntyre P. Gessner M. Dudgeon D. Prusevich A. Green P.. (2010). Global threats to human water security and river biodiversity. Nature 467, 555–561. doi: 10.1038/nature09440 20882010

[B180] Wang Y. Wang Y. Zhao J. (2022). MGA-YOLO: alightweight one-stage network for apple leaf disease detection. Front. Plant Sci. 13. doi: 10.3389/fpls.2022.927424 PMC9441945 36072327

[B181] Westbrook J. W. James J. B. Sisco P. H. Frampton J. Lucas S. Jeffers S. N. (2019a). Resistance to *Phytophthora cinnamomi* in American chestnut (*Castanea dentata*) backcross populations that descended from two Chinese chestnut (*Castanea mollissima*) sources of resistance. Plant Dis. 103, 1631–1641. doi: 10.1094/PDIS-11-18-1976-RE 31033400

[B182] Westbrook J. W. Resende M. F. R. Munoz P. Walker A. R. Wegrzyn J. L. Nelson C. D.. (2013). Association genetics of oleoresin flow in loblolly pine: discovering genes and predicting phenotype for improved resistance to bark beetles and bioenergy potential. New Phytol. 199, 89–100. doi: 10.1111/nph.12240 23534834

[B183] Westbrook J. W. Zhang Q. Mandal M. K. Jenkins V. E. Barth L. E. Jenkins J. W.. (2019b). Optimizing genomic selection for blight resistance in American chestnut backcross populations: a trade-off with American chestnut ancestry implies resistance is polygenic. Evol. Appl. 13, 31–47. doi: 10.1111/eva.12886 31892942 PMC6935594

[B184] Wipfli M. S. (1997). Terrestrial invertebrates as salmonid prey and nitrogen sources in streams: Contrasting old-growth and young-growth riparian forests in southeastern Alaska, USA. Can. J. Fish. Aquat. Sci. 54, 1259–1269. doi: 10.1139/f97-034

[B185] Wipfli M. S. Musslewhite J. (2004). Density of red alder (*Alnus rubra*) in headwaters influences invertebrate and detritus subsidies to downstream fish habitats in Alaska. Hydrobiologia 520, 153–163. doi: 10.1023/B:HYDR.0000027734.95586.24

[B186] Woodward G. Gessner M. O. Giller P. S. Gulis V. Hladyz S. Lecerf A.. (2012). Continental-scale effects of nutrient pollution on stream ecosystem functioning. Science 336, 1438–1440. doi: 10.1126/science.1219534 22700929

[B187] Zamora-Ballesteros C. Haque M. M. U. Diez J. J. Martín-García J. (2017). Pathogenicity of *Phytophthora alni* complex and P. plurivora in *Alnus glutinosa* seedlings. For. Pathol. 47, e12299. doi: 10.1111/efp.12299

[B188] Zaspel I. Naujoks G. Krüger L. Pham L. H. (2014). Promotion of resistance of black alder clones (*Alnus glutinosa* (L.) Gaertn.) against Phytophthora alni ssp. *alni* by cyclolipopeptide producing bacteria. Silvae Genet. 63, 222–229. doi: 10.1515/sg-2014-0028

